# Automated identification of leukocyte subsets improves standardization of database-guided expert-supervised diagnostic orientation in acute leukemia: a EuroFlow study

**DOI:** 10.1038/s41379-020-00677-7

**Published:** 2020-09-30

**Authors:** Ludovic Lhermitte, Sylvain Barreau, Daniela Morf, Paula Fernandez, Georgiana Grigore, Susana Barrena, Maaike de Bie, Juan Flores-Montero, Monika Brüggemann, Ester Mejstrikova, Stefan Nierkens, Leire Burgos, Joana Caetano, Giuseppe Gaipa, Chiara Buracchi, Elaine Sobral da Costa, Lukasz Sedek, Tomasz Szczepański, Carmen-Mariana Aanei, Alita van der Sluijs-Gelling, Alejandro Hernández Delgado, Rafael Fluxa, Quentin Lecrevisse, Carlos E. Pedreira, Jacques J. M. van Dongen, Alberto Orfao, Vincent H. J. van der Velden, EuroFlow Consortium, J. J. M. van Dongen, W. M. Bitter, B. R. Lubbers, C. I. Teodosio, M. Zlei, A. J. van der Sluijs-Gelling, F. de Bie, S. de Bruin-Versteeg, M. van der Burg, M. W. Schilham, V. H. J. van der Velden, A. W. Langerak, J. te Marvelde, A. E. Bras, J. Schilperoord-Vermeulen, R. Jugooa, K. C. Heezen, A. Orfao, J. Almeida, M. B. Vidriales, J. Flores-Montero, M. Pérez-Andrés, S. Matarraz, L. Martín, Q. Lecrevisse, J. J. Pérez-Morán, N. Puig, A. Medina Almeida, M. Gomes da Silva, T. Faria, M. Brüggemann, M. Ritgen, M. Szczepanowski, S. Kohlscheen, A. Laqua, E. Harbst, J. Finke, V. Asnafi, L. Lhermitte, E. Duroyon, J. Trka, O. Hrusak, T. Kalina, E. Mejstrikova, M. Novakova, D. Thurner, V. Kanderova, T. Szczepanski, L. Sędek, J. Bulsa, L. Slota, J. Kulis, C. E. Pedreira, E. Sobral da Costa, S. Nierkens, A. de Jong, A. de Koning, M. Lima, A. H. Santos, S. Böttcher, S. Lange, R. Engelmann, D. Paape, C. Machka, G. Gaipa, C. Burracchi, C. Bugarin, E. Lopez-Granados, L. del Pino Molina, L. Campos-Guyotat, C. Aanei, J. F. San Miguel, B. Paiva, L. Burgos, N. Villamor-Casas, L. Magnano, J. Philippé, C. Bonroy, B. Denys, A. Willems, P. Breughe, J. de Wolf, A. E. Sousa, S. L. Silva, P. Fernandez, D. Morf

**Affiliations:** 1 Institut Necker-Enfants Malades, Institut National de Recherche Médicale U1151, Laboratory of Onco-Hematology, Assistance Publique-Hôpitaux de Paris, Hôpital Necker Enfants-Malades, Université de Paris, Paris, France; 2 FACS/Stem Cell Laboratory, Kantonsspital Aarau, Aarau, Switzerland; 3 Cytognos SL, Salamanca, Spain; 4 Translational and Clinical Research Program, Cancer Research Centre (IBMCC, CSIC-USAL), Cytometry Service, NUCLEUS, Salamanca, Spain; 5 Department of Medicine, University of Salamanca (USAL), Salamanca, Spain; 6 Institute of Biomedical Research of Salamanca (IBSAL), Salamanca, Spain; 7 Biomedical Research Networking Centre Consortium of Oncology (CIBERONC), Instituto de Salud Carlos III, Madrid, Spain; 8 Department of Immunology, Laboratory for Medical Immunology, Erasmus MC, University Medical Center Rotterdam, Rotterdam, The Netherlands; 9 Department of Hematology, University of Schleswig-Holstein, Campus Kiel, Kiel, Germany; 10 Department of Pediatric Hematology and Oncology, University Hospital Motol, Charles University, Prague, Czechia; 11 Princess Máxima Center for Pediatric Oncology, Utrecht, The Netherlands; 12 Applied Medical Research Center (CIMA), IDISNA, Clinica Universidad de Navarra (UNAV), Pamplona, Spain; 13 Hemato-Oncology Laboratory, Portuguese Institute of Oncology, Lisbon, Portugal; 14 Tettamanti Research Center, Pediatric Clinic University of Milano Bicocca, Monza, MB Italy; 15 Pediatrics Institute IPPMG, Faculty of Medicine, Federal University of Rio de Janeiro, Av. Horacio Macedo, Predio do CT, CEP, Rio de Janeiro, 21941-914 Brazil; 16 Department of Microbiology and Immunology, Medical University of Silesia in Katowice, Zabrze, Poland; 17 Department of Pediatric Hematology and Oncology, Medical University of Silesia in Katowice, Zabrze, Poland; 18 Laboratory of Hematology, University Hospital of Saint-Etienne, Saint-Etienne, France; 19 Department of Immunohematology and Blood Transfusion (IHB), Leiden University Medical Center (LUMC), Leiden, The Netherlands; 20 Systems and Computing Department (PESC), COPPE, Federal University of Rio de Janeiro (UFRJ), Rio de Janeiro, Brazil; 21 Department of Pediatrics, Leiden University Medical Center, Leiden, The Netherlands; 22 Hemato Oncology Laboratory, Instituto Portugués de Oncologia, Lisbon, Portugal; 23 Department of Hematology, Cytometry Lab, Centro Hospitalar do Porto/University of Porto, Porto, Portugal; 24 Abteilung für Hämatologie, Onkologie und Palliativmedizin, Medizinische Klinik III, Zentrum für Innere Medizin, Universitätsmedizin Rostock, Rostock, Germany; 25 Clinical Immunology Department, University Hospital La Paz—IdiPAZ, Madrid, Spain; 26 Unitat d’Hematopathologia, Hospital Clínic de Barcelona, Barcelona, Spain; 27 Laboratory of Clinical Biology, University Hospital Ghent, Ghent, Belgium; 28 Institute of Molecular Medicine, University of Lisbon, Lisbon, Portugal

**Keywords:** Leukaemia, Laboratory techniques and procedures

## Abstract

Precise classification of acute leukemia (AL) is crucial for adequate treatment. EuroFlow has previously designed an AL orientation tube (ALOT) to guide toward the relevant classification panel and final diagnosis. In this study, we designed and validated an algorithm for automated (database-supported) gating and identification (AGI tool) of cell subsets within samples stained with ALOT. A reference database of normal peripheral blood (PB, *n* = 41) and bone marrow (BM; *n* = 45) samples analyzed with the ALOT was constructed, and served as a reference for the AGI tool to automatically identify normal cells. Populations not unequivocally identified as normal cells were labeled as checks and were classified by an expert. Additional normal BM (*n* = 25) and PB (*n* = 43) and leukemic samples (*n* = 109), analyzed in parallel by experts and the AGI tool, were used to evaluate the AGI tool. Analysis of normal PB and BM samples showed low percentages of checks (<3% in PB, <10% in BM), with variations between different laboratories. Manual analysis and AGI analysis of normal and leukemic samples showed high levels of correlation between cell numbers (*r*^2^ > 0.95 for all cell types in PB and *r*^2^ > 0.75 in BM) and resulted in highly concordant classification of leukemic cells by our previously published automated database-guided expert-supervised orientation tool for immunophenotypic diagnosis and classification of acute leukemia (Compass tool). Similar data were obtained using alternative, commercially available tubes, confirming the robustness of the developed tools. The AGI tool represents an innovative step in minimizing human intervention and requirements in expertise, toward a “sample-in and result-out” approach which may result in more objective and reproducible data analysis and diagnostics. The AGI tool may improve quality of immunophenotyping in individual laboratories, since high percentages of checks in normal samples are an alert on the quality of the internal procedures.

## Introduction

Acute leukemias (AL) represent malignant expansions of aberrant hematopoietic precursor cells arrested at an immature stage of differentiation. Current World Health Organization (WHO) classification categorizes AL on the basis of the lineage of the precursor cells and of additional morphological/cytogenetic/molecular characteristics [1]. Two major categories of AL are recognized: (1) precursor lymphoid neoplasms, which are further subdivided into B- and T-cell precursor acute lymphoblastic leukemias (BCP-ALL and T-ALL, respectively) [2] and (2) acute myeloid leukemia (AML) and related precursor neoplasms [3]. Appropriate identification of these disease categories is clinically essential as they significantly differ in therapeutic management as well as prognosis.

Flowcytometric immunophenotyping is a frontline diagnostic tool of AL used to identify the lineage of leukemic cells and subsequently orientate the therapeutic strategy. While lineage assignment is relatively straightforward in most cases (~95%), leukemic cells may on rare occasions (~5%) either express differentiation antigens specific to more than one lineage—mixed phenotype AL (MPAL)—or show no clear evidence of differentiation along a single lineage—acute undifferentiated leukemia (AUL). These cases are gathered in a separate category (AL of ambiguous lineage) in the current WHO classification [4–6].

EuroFlow recently developed databases of fully annotated flow cytometry data files from normal and pathological samples stained with EuroFlow screening tubes and antibody panels, particularly for minimal residual disease (MRD) monitoring in multiple myeloma and BCP-ALL, and for diagnosis of primary immune deficiencies and immune monitoring [7–11]. Here, we report on the development of a standardized strategy for initial assessment of precursor cells in AL diagnostic samples (B- or T-lymphoid vs. non-lymphoid lineage or mixed phenotype) in order to allow appropriate orientation toward complementary BCP-ALL, T-ALL, and/or AML/MDS antibody classification panels [10]. This was based on a single eight-color tube called AL orientation tube (ALOT) analyzed according to the EuroFlow standardized operating procedures (SOP) released in 2012 [12]. We recently reported on the validation of a large ALOT database and the development of an automated database-guided analytical algorithm to support subsequent selection of the appropriate classification panel(s) for the diagnosis of AL patients (Compass tool) [13]. This work demonstrated that standardized diagnostic procedures could allow comparison of samples with a reference database for fast and robust orientation of the samples to the appropriate diagnostic panel. Such strategy is even more attractive as it enables integration of multiple immunophenotypic characteristics at the single-cell level to fully describe the cellular subsets of a leukemic sample, and thereby, to get accurate insight into the phenotypic heterogeneity of AL which could improve the diagnosis and management of AL in the near future [13].

Since this is a single-cell-based approach, it is highly sensitive to the quality of the population selection criteria, i.e., the quality of the gating. Thus, automated gating strategies emerge as very attractive to include the gating procedure within the standardization process, speed up the analysis and improve the selection of abnormal cells to be further classified [14–20]. Previously, an automated gating and identification (AGI) tool was designed within the consortium and successfully validated with, e.g., the lymphoid screening tube for which a dedicated database was built [21]. In the current study, we built a reference database of normal peripheral blood (PB) and bone marrow (BM) samples stained with the previously validated ALOT antibody combination. This database served as a reference for accurate, reproducible and AGI of distinct normal and different from normal cell subsets contained in independent ALOT data files [21]. We developed a pipeline combining this AGI tool with the database-guided algorithm to minimize human intervention and improve standardization. Normal BM and PB samples were used to evaluate how the AGI tool performs, and then leukemic samples were analyzed by experts and the AGI tool in parallel to compare the two approaches. The AGI tool classified each and every single event from tested samples into either normal populations or the so-called “checks” that designates events that could not be classified with a high probability into normal subsets and required a review by an expert for correct final assignment. After review by the expert, the abnormal population served as an input to the database-guided algorithm which used the Compass tool to describe the phenotypic content of the abnormal population, report its composition and propose the diagnostic(s) panel(s) to be performed. Here, we report on the results of this pipeline which demonstrated strong reliability in delineation of normal vs. abnormal cells and the validation of our database-guided software-assisted strategy, for (semi-)automated analysis of ALOT data.

## Material and methods

### Construction of the ALOT database for PB samples

**Fig. 1:**
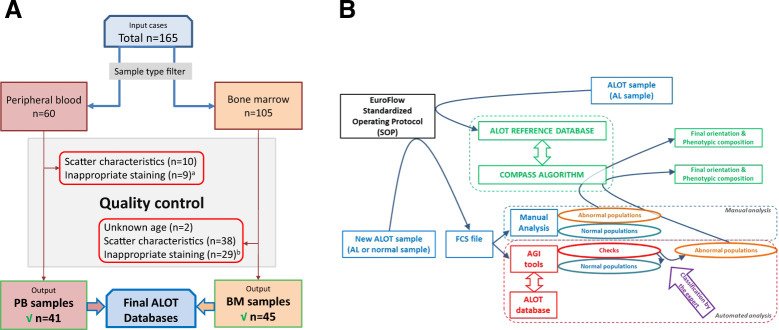
Overview of the study. **a** Construction of the ALOT databases. ^a^smCD3 or cyCD3 (*n* = 8), general (*n* = 1); ^b^smCD3 or cyCD3 (*n* = 5), CD19 (*n* = 2), cyCD79a (*n* = 3), MPO (*n* = 18); CD45 (*n* = 3); CD7 and CD34 (*n* = 2). **b** Overall pipeline for data analysis of tested samples. An ALOT database of flow cytometry data files corresponding to normal PB and BM samples stained with ALOT was built to serve as a reference to use in combination with the automated gating and identification (AGI) tool (red box). Any leukemic sample analyzed using the ALOT and the EuroFlow standardized operating protocol (SOP) could serve as an input to the AGI tool (left panel). Every single event was assigned to normal populations according to the reference database. Events whose immunophenotypic pattern did not match the exact phenotype of normal populations were labeled as “checks” and were submitted to the expert for appropriate final classification into debris, doublets, normal subsets, or abnormal population. Abnormal (leukemic) population(s) were then processed by our previously published database-guided expert-supervised algorithm (Compass tool) to describe the phenotypic composition of the leukemic population(s) and guide toward the appropriate panel for complete characterization and diagnosis of the leukemia subtype (green box). Briefly, this process used a compass algorithm and a large ALOT reference database of 656 leukemic samples to compare the immunophenotypic patterns and provide an output. To evaluate the performance of the AGI tool, a manual analysis was run, followed by the Compass algorithm in parallel to this AGI and Compass analyses. Comparison of the two approaches was based on: the number and nature of checks, the phenotypic description of the leukemia bulk, and the final panel orientation as readouts.

A total of 60 normal PB samples were available for construction of the PB database, originating from eight EuroFlow centers. Very stringent selection criteria were applied to the samples included in the database in order to train the tool with a very neat dataset. Overall 19/60 samples (32%) were excluded because of suboptimal technical quality: mostly for inadequate light scatter and/or inadequate intracellular staining, particularly for cyCD3. Therefore, the final PB ALOT panel database contains Flow Cytometry Standard (FCS) data files from 41 normal samples (see “Results” section for details and Fig. 1a), having a gender distribution of 78% females and 22% males, and a median age of 50 years (range: 1–79 years).

### Construction of the ALOT database for BM samples

A total of 105 BM samples were available for construction of the BM database, originating from eight EuroFlow centers. The same stringent selection criteria used for PB samples were applied and 60 samples (57%) were excluded because of suboptimal technical quality: inappropriate light scatter when compared to other samples from the same center and among centers, and/or inappropriate staining, particularly for MPO, that in some cases did not allow clear separation between neutrophils and monocytes. Therefore, the final BM ALOT panel database contains FCS data files from 45 normal samples (see “Results” section for details and Fig. 1a): 13 samples for the age group of <5 years, 11 samples for the age group 6–15 years, and 21 samples for the age group of ≥16 years.

### Gating strategy of normal cases included in the database

For the analysis of the FCS data files to be included in the database, a special gating strategy was used. The process of gating is exemplified in Supplementary Fig. 1 (for PB) and Supplementary Fig. 2 (BM), and extensively detailed in the Supplementary information. Before building the final databases, each individually identified BM cell population was compared among the distinct age-ranges using multidimensional principal component (PC) 1 vs. PC2 analysis, to confirm there were no immunophenotypic differences (<2.5 SD) between the age groups (Supplementary Fig. 3).

### Automated gating and identification tool

The AGI tool is included in the Infinicyt software [7, 21, 22]. To validate the ALOT databases the Infinicyt software—version 2.0 release candidate (RC) 33 (Infinicyt 2.0.0c RC33) was used. As general principle, the algorithms included in the AGI tool work in two steps: (1) unsupervised, multidimensional clustering with parameters configurable for each panel for ALOT a minimum number of events (K) per cluster of 10 and a maximum multidimensional distance (S) of 0.9 were used) and (2) classification of cell populations, in which each cluster is plotted using multidimensional canonical correlation analysis (CA) against each cell population in the database. Then, the result of these comparisons is translated into a population tree containing groups of clusters joined under the same label, with 100% certainty (normal cell populations) or with doubts (clusters that are similar to a database group, but not inside the 2.5 SD of the reference cell populations included in the database, the latter being required to be checked more carefully by an expert.

An important part of the AGI tool is the number and type of alarms corresponding to each age-range, allowing the user to identify cases with blasts having aberrant as well as normal phenotypes but at abnormal frequencies.

The ALOT AGI tool is connected with a classification database [13] in such a way that once the expert assigns an abnormal population, this is automatically classified by applying the Compass tool and offering a result pointing to the most probable disease category and required AL classification panel(s).

### Reporting on results

To speed up the analysis, normalize the interpretation of the data and facilitate the expert review, an automated report tool was designed to summarize the sample features (e.g. quantitative distributions out-of-range vs. database and description of expression of the ALOT markers for the abnormal cell population). The process for summarizing data is detailed in the Supplementary.

### Samples used to validate the AGI tool

Samples were collected after written informed consent had been given according to the Declaration of Helsinki. Samples were then processed and stained locally at each center, using EuroFlow SOPs for sample preparation and staining, and data acquisition [12]. Samples stained with the ALOT were measured on FACSCanto II flow cytometers (BD Biosciences, San Jose, CA), calibrated and monitored according to the EuroFlow SOP for instrument set-up [12]. Sample preparation, instrument settings, and staining protocols are available at www.euroflow.org. The study was approved by the local ethics committees of the participating centers. Characteristics of normal and patient samples are presented in Supplementary Table 3. All sample files were anonymized and coded according to an internal EuroFlow coding protocol. In contrast to those samples used for building the database, no stringent selection criteria were applied to the samples employed to validate the AGI tool and all available samples obtained using the EuroFlow protocols were included.

### Validation strategy

To validate the AGI tool for the ALOT, a workflow as described in Fig. 1b was used. Briefly, tested samples were analyzed by the AGI tool which classified each and every single event into either normal populations or the so-called “checks” that designate events that could not be properly classified into any of the normal subsets in the ALOT database and required a review by an expert for correct assignment. After review by the expert, the abnormal population served as an input to the database-guided algorithm that used the Compass tool to describe the phenotypic content of the abnormal population, report on its composition and propose the diagnostic(s) panel(s) to be subsequently performed. To evaluate the performance of this process, it was compared to our previously published manual gating strategy in which normal and abnormal populations were all identified by the expert before comparison to the reference ALOT database. The following variables were used to compare the two approaches: number of events before (pre-AGI review) and after expert review (post-AGI review), number of checks generated by the AGI tools and final orientation of the AL population by the Compass tool. Finally, we also evaluated the performance of the AGI and Compass tools using other commercially available ALOT reagent mixes (BD ALOT OneFlow and Cytognos ALOT kits).

### Reproducibility

To evaluate the intra-expert reproducibility of data obtained by manual analysis and the AGI tool, 26 data files from 26 subjects (one PB, 25 BM; five normal, three T-ALL, six B-ALL, nine AML, and three MPAL) were analyzed by seven experts by both manual analysis as well as the AGI tool. In addition, to evaluate inter-expert reproducibility ten data files (four normal, three AML, three B-ALL) were independently analyzed for a second time by three experts. Event numbers of the various populations were recorded. In addition, for calculation of mean and %CV values, all data with <50 events (i.e., outside the quantitative range of the flow assay) were excluded. Mean and %CV values were calculated for each of the 26 samples for the identified cell populations.

## Results

### Database construction

Reference databases were built independently for PB and BM samples. For this process, a crucial step of strict case selection was performed, summarized in Fig. 1a. Very stringent selection criteria were applied to the samples included in the database in order to train the AGI tool with a very neat dataset. Out of the initial 165 normal/reactive samples (60 PB and 105 BM), 86 samples (41 PB and 45 BM) were finally included in the databases. The main reason for sample exclusion was suboptimal technical quality (*n* = 19 PB and *n* = 60 BM samples).

After the strict case selection, each individual case was analyzed according to a predefined and proven-reproducible gating strategy (Supplementary Figs. 2 and 3 for PB and BM, respectively). The result of such analysis was a completely analyzed FCS data file, with all events assigned to a particular population, including cell doublets and debris. The number of populations identified was different between BM (*n* = 35) and PB (*n* = 37), mostly due to the presence of specific cell populations in BM which are absent in PB (e.g., CD34+ B-cell precursors). All individual analyzed data files generated belonging to the same sample type (BM or PB) were then merged and saved as final BM and PB ALOT reference databases.

Thus, both ALOT reference databases consisted of digital libraries of FCS data, containing information about: (1) the events of all cell populations from individual cases; (2) pre-calculated data on comparisons among these cell populations required by the *n*-dimensional algorithms used in the classification phase (e.g. for PB and BM a total of 1332 and 1190 two-group canonical analysis comparatives were stored, respectively); (3) descriptive statistical data at both the population and parameter levels (to be used for alerting on deviations from normal later on, in the process of AGI); (4) the configuration of the sensitivity and specificity parameters for the clustering algorithms; (5) a set of official configurations called a profile containing all details required for the final output (e.g., diagrams, population tree, alerts, report); (6) a superior number of identified and stored populations compared to the final output, due to the specific requirements of the classification algorithms, but that are of no relevance or interest for the user (e.g., subdivision of debris and doublets).

### Validation of the AGI tool with normal samples

**Fig. 2:**
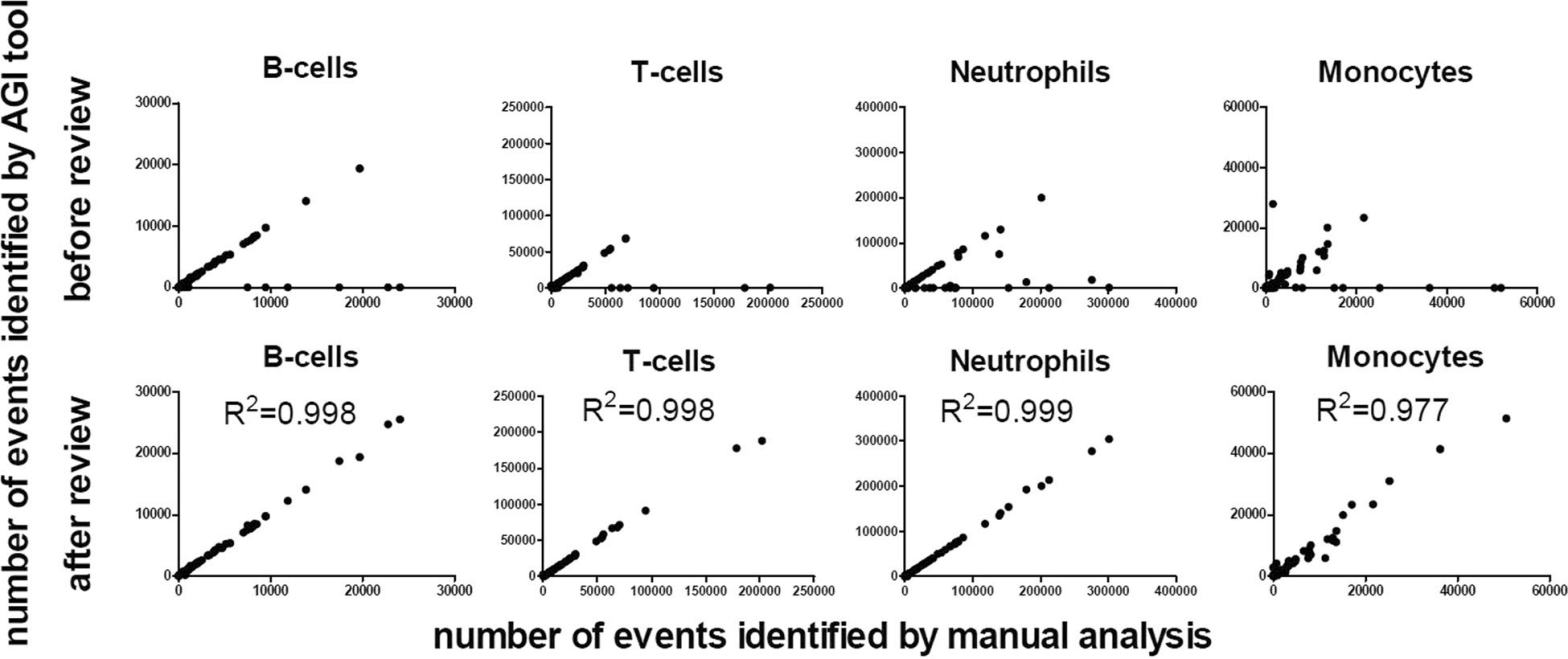
Correlations between the number of events for different leukocyte subsets present in peripheral blood as analyzed by manual analysis vs. the AGI tool. Pearson *R*^2^ are shown. Post-AGI review, all correlations had *p* values <0.001.

**Fig. 3:**
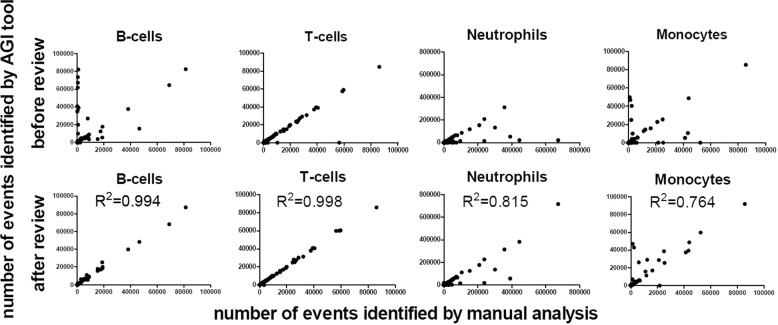
Correlations between number of events for different leukocyte subsets present in bone marrow as analyzed by manual analysis vs. the AGI tool. Pearson *R*^2^ are shown. Post-AGI review, all correlations had *p* values <0.001.

To validate the performance of the AGI tool normal samples acquired using the ALOT were first used (Fig. 1b). Normal PB samples (*n* = 43) were analyzed both manually and with the AGI tools for recognition of normal B, NK, and T cells, as well as neutrophils, eosinophils, and monocytes, based on the ALOT antibody combination and light scatter. The frequency of checks with the AGI tools ranged from 0.04 to 88% (median: 2%), with highest frequency of checks for neutrophils and monocytes. Interestingly, the number of checks appeared to be highly dependent on the center generating the data (mean of 0–67% checks; Supplementary Fig. 4), reflecting the local adherence to SOP requirements, rather than the performance of the AGI tools. Number of events per population were generally highly similar between manual and automated analysis before review of the checks by the expert (pre-AGI review), except for a small subset of samples (Fig. 2, top row). The discrepancy tended to be higher in myeloid vs. lymphoid populations and it was consistently related to classification of events into checks, so that subsequent review by the expert (post-AGI review) led to even higher correlation of both analytical methods in terms of number of events (Fig. 2, bottom row). Altogether, post-AGI coefficients of correlation (*r*^2^) were constantly >0.990 suggesting that the AGI tool and manual analysis performed with nearly identical results in PB samples. In order to rule out a sample effect, the same comparisons were repeated on 31 normal BM samples. This showed similar results with higher correlation for the number of events after expert review (post-AGI review) (Fig. 3). Also, in BM best concordance was obtained for lymphoid cells. Although, the more heterogeneous myeloid populations showed greater variability between manual analysis and AGI, good concordance was still observed (Fig. 3). Number of checks in BM was overall slightly higher than in PB but systematically <10%, possibly related to a higher frequency of (and more heterogeneous) myeloid compartments.

Once the approach had been validated with samples stained with the reference (liquid format) EuroFlow reagents, the performance with other commercially available (dried format) reagents (i.e., BD OneFlow (www.bdbiosciences.com) and Cytognos (https://www.cytognos.com) reagents) was evaluated. Again, a high concordance in the number of events for the distinct normal cell populations identified with ALOT using both manual and automated analysis was observed irrespective of the sample nature—Supplementary Fig. 5 (BD OneFlow) and Supplementary Fig. 6 (Cytognos kit). Moreover, the frequency of checks was also similar for the different ALOT reagents and remained <3% in PB, and <10% in BM (Supplementary Figs. 7 and  8). The only exception was the identification of neutrophils, which on average showed a higher number of checks when the BD OneFlow reagent (BM samples) and the Cytognos kit (PB samples) were used.

### Validation of the AGI tool with leukemic samples

Subsequent evaluation of the AGI tool using AL samples (Supplementary Table 3) showed alarms for expert review in 96/96 Al samples. In 91/96 cases (95%), leukemic cells were assigned to the checks population, while in five patients AL cells were assigned to a normal population (CD34+ myeloid precursors in two AML cases, monocytes in two AML cases, and CD34+ B-cell precursors in one BCP-ALL case), with numerical alarms in the later cases. Leukemic cell counts in both PB and BM samples showed a high correlation between manual and automated analysis (Supplementary Fig. 9).

**Fig. 4:**
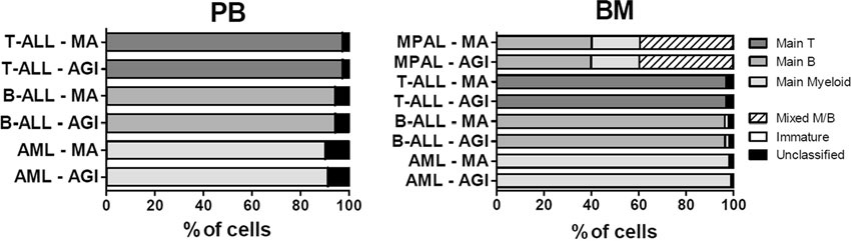
Comparison of Compass tool data for acute leukemia cells in peripheral blood (PB, left) or bone marrow (BM, right), as identified by manual analysis (MA) or the AGI tool (AGI). Representative examples are shown, additional cases are shown in the Supplementary materials.

Subsequently, we analyzed the final diagnostic orientation provided by the Compass tool based on the results of manual vs. automated data analysis, including the detailed phenotypic composition provided by the two approaches. In 13 AML, 11 BCP-ALL and 10 T-ALL PB samples a high concordance between the two methods in terms of phenotypic composition was observed (Supplementary Fig. 10a–c) with a final 100% concordance (i.e., comparable arrows in the Compass plot and similar direction to subsequent classification panel(s)). To confirm these results on BM samples, we studied 19 AML, 27 BCP-ALL, 10 T-ALL, and 5 MPAL samples. Once again a high concordance between the two data analysis approaches was observed, with only subtle variations in the phenotypic composition of the samples (Supplementary Fig. 11), except for one case (B-ALL4); in this latter case, detailed evaluation showed the discrepancy was due to the presence of doublets and/or dead cells in the final ALL population as defined after AGI tool, which had been excluded by manual analysis. Altogether, both manual and automated analyses resulted in very similar descriptions of the leukemic cell contents and phenotypes (representative examples are shown in Fig. 4), with full concordance in the final diagnostic orientations for every case evaluated.

### Reproducibility of expert-based manual analysis vs. automated analysis

**Fig. 5:**
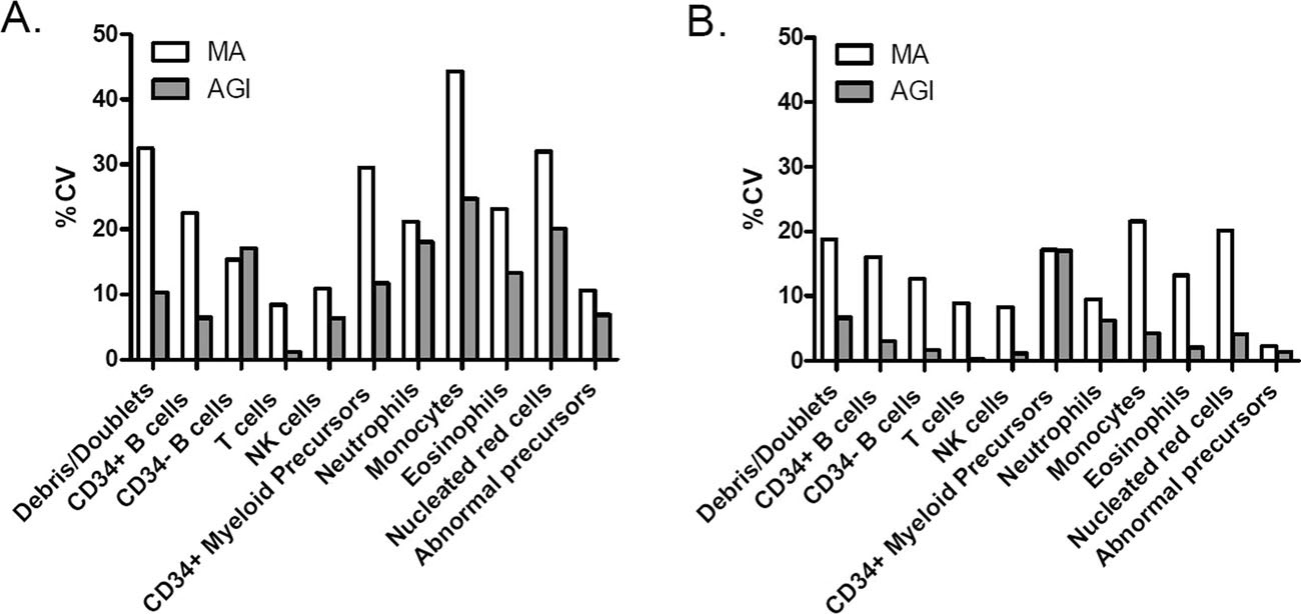
Reproducibility of manual analysis (MA) and AGI analysis. **a** Twenty-six samples were evaluated both manually and by AGI tool by seven independent experts to define the intra-person variability. **b** Ten samples were independently analyzed twice by three experts both manually and by AGI tool to define the inter-person variability. Data represent mean values of %CV values calculated for each of the 10 samples for the indicated cell populations.

Flowcytometry is often challenged as being a highly subjective, expert-dependent technique, with limited reproducibility across multiple centers [23]. Introduction of automated analysis via construction of a database of reference samples analyzed by recognized experts might allow for significantly higher levels of reproducibility by applying the same rules for classification of single events into cell subsets as if samples had all been analyzed by the same expert. We compared the reproducibility of results obtained in 26 data files from 21 AL patients and five healthy individuals analyzed by seven EuroFlow experts with both the AGI tool and manual analysis. As shown in Fig. 5, automated analyses led to considerably lower intra-expert and inter-expert variability as compared to manual analysis, indicating that automated analysis is able to improve reproducibility of FCS analyses.

## Discussion

Recently, we reported on the development and validation of an automated database-guided analytical algorithm in combination with a large ALOT database to support subsequent selection of the appropriate classification panel(s) for the diagnosis of AL [13]. This work demonstrated for the first time that standardized (automated) diagnostic procedures based on comparison of single-cell phenotypes from AL samples to a reference database, provides fast and robust orientation to the most appropriate AL classification panel. However, results were highly dependent on the manual gating strategy used to identify the abnormal/leukemic cell populations, thereby introducing a level of subjective expertise in the system [23]. A major benefit of the novel database-guided analysis was the use of single-cell data for classification of events, by providing insight into the detailed composition of the leukemic samples rather than the overall characterization of the bulk cell population. Such deep characterization of AL samples at the single-cell level is obviously hampered by a population-based gating of blast cells performed by the expert, which might frequently include variable proportions of contaminating cells [13]. Thus, by adding a single-cell-based automated analysis tool upstream of the database-guided classification of leukemic events might contribute to improve the global data analysis approach by providing optimal input to the system and getting the best results out of the Compass tool. Fast and appropriate classification of single cells based on integration of the whole immunophenotype of single-cell events would indeed result in (more) objective gating and accurate exclusion of possible contaminants that an expert cannot easily eliminate.

The AGI tool recognized virtually all AL cells as being not-normal. Thus, these cells were generally (~95%) included in the checks population with an alarm indicating that the number of checks was higher than expected for normal PB and BM samples. This demonstrates that even with a single eight-color tube, leukemic cells can already be distinguished from normal cells in virtually every AL case. In only a few cases (~5%), AL cells appeared to have an immunophenotype identical to that of normal cells for the eight markers contained in the ALOT; consequently, in these cases, blasts were included in one of the normal cell population but with a clear numerical alarm that inform/alarm the expert for a need to review the abnormally increased “normal” cells and allocate them to the leukemic population. Thus, both qualitative criteria (phenotypic checks) and quantitative criteria (frequency checks) were important and taken into account to create an alert. Importantly, in all cases evaluated, the AL cells were identified with either the “checks” label or a numerical alarm.

Number of events, phenotypic composition, and final orientation to the appropriate diagnostic panel were highly concordant between manual and automated analyses irrespective of the sample nature (PB or BM). This confirms that the AGI tool performs well. Whenever minor differences were observed in terms of population frequency or phenotypic composition, they never had an impact on the final diagnostic orientation to subsequent AL characterization panels. Importantly, inter-operator variability of AGI tool-derived data was never higher than data obtained by manual analysis by several well-trained experts within the EuroFlow consortium. Inter-operator variability will likely be larger when samples are manually analyzed by operators that are less experienced [11], emphasizing the beneficial effect of the AGI tool in real-world clinical flow cytometry laboratory diagnostics. Our approach certainly results in consistent reliability and high level of reproducibility comparable to what can be obtained by a group of experienced flow cytometry experts, thereby providing a better degree of confidence to less-experienced cytometrists. We did not specifically evaluate repeated analysis by the same operator at multiple times, but we cannot preclude this would have led to a greater variability, vs. computer-assisted gating using AGI which has already been shown not to be influenced by time [11]. As a consequence, it is quite clear that automated gating significantly improves the reproducibility and robustness of the gating procedure.

Another major advantage of this automated gating approach in the laboratory diagnostics settings is the increased number of checks observed whenever deviations from the standard technical recommendations (e.g., SOPs) occur (e.g., by alarming for a missing reagent, unstable instrument flow, incomplete red cell lysing, or suboptimal antibody titers and incubation time) [12, 13]. Thus, while the overall number of checks in normal PB and BM was minimal, it varied significantly among distinct laboratories. In line with this, check events were virtually absent in some centers, while significantly higher in other laboratories. In addition, experience of the EuroFlow group suggests that appropriate training could help in reducing the number of checks by improving adherence to SOPs and correcting deviations from the protocol. These results confirm that the percent alarmed events provided by the here proposed automated gating approach, not only reflects how the AGI tool performs, but it also provides indication about how laboratories perform, setting the basis for innovative and more accurate (multicentric database-based) external quality control tools for flow cytometry laboratories, applicable to every ALOT sample run in the flowcytometer. Overall, this also indicates that despite <3% checks in normal PB and <10% checks in normal BM might be acceptable, they can be locally decreased in some laboratories, which would further enhance the benefit of automated gating by minimized human intervention. In any case, situations of check overload should be considered as an alert about the quality of the internal procedures, which prompts individual centers to review and optimize SOP compliance to improve the quality. Previous experience within EuroFlow demonstrated that subsequent training of centers with higher percentage of checks/alarms show decrease number of alarms. Strong compliance to the instrument setup and sample processing SOPs are thereby of utmost importance for appropriate use of both the AGI tools and database-guided diagnostic orientation toward the most appropriate subsequent AL panel, and should be verified with already existing quality controls and the local average number of checks [24–27]. Of note, the use of validated alternative reagents, like the OneFlow ALOT and the Cytognos ALOT kits, resulted in highly comparable results. This provides the user with broader reagent selection possibilities, without an impact on the automated gating and diagnostic orientation results.

Despite all the above, the number of checks observed in our study was also influenced by the population evaluated. Thus, more checks were observed for myeloid and monocytic populations than in the lymphoid compartments. This is most likely related to the fact that the ALOT includes numerous lymphoid markers (CD45, smCD3, cyCD3, CD7, cyCD79a, CD19) with more limited value of light scatter (FSC, SSC) for identification of lymphoid populations, while few markers are present for gating of myeloid/monocytic cells (cyMPO, CD45) and their gating strongly relies on light scatter which are strongly dependent on adherence to the sample preparation SOPs for staining of intracellular markers. Indeed, it is commonly accepted that, compared to fluorescence measurements, quantitation of light scatter is more difficult to standardize [12, 24, 25]. Despite this, data were never compromised by variations in scatter characteristics and AGI data never inappropriately identified the leukemic population.

Overall, it is important to emphasize that the aim of evaluating also the compass tool was not to argue whether the final diagnosis was right or not, because the ALOT does not provide a final diagnosis. It is a validated and approved orientation tube [10] that points toward the characterization panel that should be run for precise and definitive characterization of the AL and final diagnosis, as previously addressed elsewhere [13]. In contrast, our goal was to show that data on the leukemic cells identified by either manual analysis or AGI are highly comparable and result in similar conclusions regarding subsequent protocols. Keeping with this, all samples included in the study were either normal or AL samples with prior cytomorphological confirmation of the specific AL diagnosis. Other samples such as myelodysplastic syndromes with excess in blasts or mature lymphoid malignancies were not included as these do not represent an appropriate indication of use of the ALOT alone. Other panels were designed for these specific situations and should be run in as previously indicated [10]. In addition, the ALOT was originally designed to analyze samples with a clinical and cytomorphological suspicion of AL and (relatively) significant infiltration of, e.g., BM (>5%) [10]. Even though our results seem to show high performance to appropriately classify rare events, we strongly recommend using our strategy with respect of this mandatory requirement. We have no data in this study to support that such an approach could be reliably used for MRD assessment and the configuration of the ALOT is not appropriate for such an evaluation. This will have to be tested with appropriate MRD antibody panels [8] in an independent study, which is beyond the scope of the present work.

Another major advantage of the whole AGI-Compass pipeline relies on the ability to generate an automated report that includes all the clinically relevant information related to the presence (vs. absence) and immunophenotype of the abnormal population(s) and panel orientation (see example in Supplementary Fig. 12). All-in-all, this results in a highly automated process in which the operator takes part at two stages: (1) to appropriately classify the checks that the algorithms could not assign with sufficient confidence according to the reference database; (2) to review the conclusions by comparing the automated interpretation with human expertise, before drawing final clinical conclusions. Generating an automated report that can be edited, offers a unique opportunity to speed up the process and provide at glance extended immunophenotypic information to the clinician, particularly if checking of unclassified events is minimized by strictly controlling the quality of the sample and the laboratory SOPs.

Altogether, here we propose and validated a new flow cytometry tool that represents an innovative step in minimizing human intervention and requirements for expertise, toward a simplified and more robust “sample-in and result-out” automated approach that can be used with both the reference (liquid format) and alternative commercially available (dried format) ALOT reagents. This highly automated pipeline has the advantage to speed up the process, and to be valuable for accreditation as it improves intra- and inter-laboratory reproducibility and thereby, the quality in individual laboratories, as long as appropriately used in combination with the EuroFlow SOPs.

## Supplementary information

Supplemental data
