# Supplementary material for: Automated identification of leukocyte subsets improves standardization of database-guided expert-supervised diagnostic orientation in acute leukemia: a EuroFlow study
Source: Mod Pathol. 2020 Sep 30;34(1):59–69. doi: 10.1038/s41379-020-00677-7 (PMC7806506; doi:10.1038/s41379-020-00677-7)
Supplement: Supplementary file 1 — Supplemental data [file 41379_2020_677_MOESM1_ESM.pdf]

Supplemental data

Automated identification of leukocyte subsets improves standardization of database-guided expert-supervised diagnostic orientation in acute leukemia, a EuroFlow study.

Ludovic Lhermitte[1], Sylvain Barreau[1#], Daniela Morf[2#], Paula Fernandez[2], Georgiana Grigore[3], Susana Barrena[3,4], Maaike de Bie[5], Juan Flores-Montero[4], Monika Brüggemann[6], Ester Mejstrikova[7], Stefan Nierkens[8], Leire Burgos[9], Joana Caetano[10], Giuseppe Gaipa[11], Chiara Buracchi[11], Elaine Sobral da Costa[12], Lukasz Sedek[13], Tomasz Szczepański[14], Carmen-Mariana Aanei[15], Alita van der Sluijs-Gelling[16], Alejandro Hernández Delgado[3,4] , Rafael Fluxa[3], Quentin Lecrevisse[4], Carlos E. Pedreira[17], Jacques J.M. van Dongen[16], Alberto Orfao[4], Vincent H.J. van der Velden[5]

[1] Université de Paris, Institut Necker-Enfants Malades, Institut National de Recherche Médicale U1151, Laboratory of Onco-Hematology, Assistance Publique-Hôpitaux de Paris, Hôpital Necker Enfants-Malades, Paris, France
[2] FACS/Stem cell Laboratory, Kantonsspital Aarau, Aarau, Switzerland
[3] Cytognos SL, Salamanca,Spain
[4] Translational and Clinical Research program, Cancer Research Centre (IBMCC, CSIC-USAL), Cytometry Service, NUCLEUS; Department of Medicine, University of Salamanca (USAL); Institute of Biomedical Research of Salamanca (IBSAL), Salamanca, Spain and Biomedical Research Networking Centre Consortium of Oncology (CIBERONC), Instituto de Salud Carlos III, Madrid, Spain.
[5] Laboratory for Medical Immunology, Department of Immunology, Erasmus MC, University Medical Center Rotterdam, Rotterdam, The Netherlands
[6] Department of Hematology, University of Schleswig-Holstein, Campus Kiel, Kiel, Germany
[7] Department of Pediatric Hematology and Oncology, University Hospital Motol, Charles University, Prague, Czechia
[8] Princess Máxima Center for Pediatric Oncology, Utrecht, The Netherlands
[9] Clinica Universidad de Navarra (UNAV), Applied Medical Research Center (CIMA), IDISNA, Pamplona, Spain
[10] Hemato-Oncology Laboratory, Portuguese Institute of Oncology, Lisbon, Portugal
[11] Tettamanti Research Center, Pediatric Clinic University of Milano Bicocca, Monza (MB) Italy
[12] Pediatrics Institute IPPMG, Faculty of Medicine, Federal University of Rio de Janeiro, Av. Horacio Macedo, Predio do CT, CEP 21941-914 Rio de Janeiro, Brazil
[13] Department of Microbiology and Immunology, Medical University of Silesia in Katowice, Zabrze, Poland
[14] Department of Pediatric Hematology and Oncology, Zabrze, Medical University of Silesia in Katowice,
[15] University Hospital of Saint-Etienne, Laboratory of Hematology, Saint-Etienne, France
[16] Department of Immunohematology and Blood Transfusion (IHB), Leiden and University Medical Center (LUMC), Leiden, the Netherlands
[17] Systems and Computing Department (PESC), COPPE, Federal University of Rio de Janeiro (UFRJ), Brazil

Supplemental information

Gating strategy of normal cases included in database

For the analysis of the FCS datafiles to be included in the database, a special gating strategy was used. Due to the specific algorithms used for automated gating and identification of FCS data, all events from a sample to be introduced in the database have to be classified, debris and doublets included. In order to assign all events to particular cell populations, the gating strategy started with large gating on lineage markers and then subsequent gating for exclusion of debris and doublets, and/or sub-classification into subpopulations -Supplemental Figure 1 (for PB) and Supplemental Figure 2 (BM)-. In both databases a specific population called unspecified nucleated cells contained all cell subsets that could not be unequivocally labeled using the ALOT combination. This cell category contained various known cell subsets (like basophils and dendritic cells) that cannot be correctly identified with ALOT. Before building the final databases, each individually identified BM cell population was compared among the distinct age-ranges using multidimensional principal component (PC) 1 vs PC2 analysis (PCA), to confirm there were no immunophenotypic differences (<2.5 SD) between the age groups (Supplemental Figure 3).

Automated gating and identification tool

The AGI tool is included in the Infinicyt software.*(1–3)* To validate the ALOT databases the Infinicyt software - version 2.0 release candidate (RC) 33 (Infinicyt 2.0.0c RC33) was used. As general principle, the algorithms included in the AGI tool work in two steps: i) unsupervised, multidimensional clustering with parameters configurable for each panel for ALOT a minimum number of events (K) per cluster of 10 and a maximum multidimensional distance (S) of 0.9 were used) and ii) classification of cell populations, in which each cluster is plotted using multidimensional canonical correlation analysis (CA) against each cell population in the database. Then, the result of these comparisons is translated into a population tree containing groups of clusters joined under the same label, with 100% certainty (normal cell populations) or with doubts (clusters that are similar to a database group, but not inside the 2.5 SD of the reference cell populations included in the database, the latter being required to be checked more carefully by an expert.

An important part of the AGI tool is the number and type of alarms corresponding to each age-range, allowing the user to identify cases with blasts having aberrant as well as normal phenotypes but at abnormal frequencies.

The ALOT AGI tool is connected with a classification database *(4)* that once the expert assigns an abnormal population is automatically applying the Compass tool and offering a result pointing to the most probable disease category and required AL classification panel(s).

Reporting on results

To speed up the analysis, normalize the interpretation of the data and facilitate the expert review, an automated report tool was designed to summarize the sample features (e.g quantitative distributions vs. normal reference ranges and description of expression of the ALOT markers for the abnormal cell population). The result of the process of automated gating and identification is a fully analyzed sample file, showing frequencies for all cell populations (normal and abnormal) in the statistics column and also in the report section of Infinicyt. For each cell population and sample type, an age-dependent reference range (0-5y, 6-15y and >16y; Supplemental Table 1 and 2) was applied, and all values out of range being underlined in the alert column in the profile and in bold font in the report. The reference ranges herein defined did not consist in universal references but were rather used to define cut-offs beyond which an alert is provided by the software to draw the attention of the operator so that the latter can focus on unusual numbers.

If an abnormal population was identified, the Compass tool was automatically performed and the resulting data shown in text format in the report. The software also provided data on the comparison of median fluorescence intensity per marker between the abnormal cells and the reference normal cells in the corresponding ALOT database and scored expression of individual markers as negative, positive low, positive, strong positive, or heterogeneous. Whenever the result was “heterogeneous expression”, a percentage of positivity was displayed. For the calculation of the percent positivity, cut-offs were defined for each parameter and each normal reference cell population (data not shown).

1. J. Flores-Montero, L. Sanoja-Flores, B. Paiva, N. Puig, O. García-Sánchez, S. Böttcher, V. H. J. van der Velden, J.-J. Pérez-Morán, M.-B. Vidriales, R. García-Sanz, C. Jimenez, M. González, J. Martínez-López, A. Corral-Mateos, G.-E. Grigore, R. Fluxá, R. Pontes, J. Caetano, L. Sedek, M.-C. Del Cañizo, J. Bladé, J.-J. Lahuerta, C. Aguilar, A. Bárez, A. García-Mateo, J. Labrador, P. Leoz, C. Aguilera-Sanz, J. San-Miguel, M.-V. Mateos, B. Durie, J. J. M. van Dongen, A. Orfao, Next Generation Flow for highly sensitive and standardized detection of minimal residual disease in multiple myeloma, *Leukemia* **31**, 2094–2103 (2017).

2. J. Flores-Montero, G. Grigore, R. Fluxá, J. Hernández, P. Fernandez, J. Almeida, N. Muñoz, S. Böttcher, L. Sedek, V. van der Velden, S. Barrena, A. Hernández, B. Paiva, Q. Lecrevisse, M. Lima, A. H. Santos, J. J. M. van Dongen, A. Orfao, EuroFlow Lymphoid Screening Tube (LST) data base for automated identification of blood lymphocyte subsets, *Journal of Immunological Methods* , 112662 (2019).

3. J. J. M. van Dongen, M. van der Burg, T. Kalina, M. Perez-Andres, E. Mejstrikova, M. Vlkova, E. Lopez-Granados, M. Wentink, A.-K. Kienzler, J. Philippé, A. E. Sousa, M. C. van Zelm, E. Blanco, A. Orfao, EuroFlow-Based Flowcytometric Diagnostic Screening and Classification of Primary Immunodeficiencies of the Lymphoid System, *Front. Immunol.* **10**, 1271 (2019).

4. L. Lhermitte, E. Mejstrikova, A. J. van der Sluijs-Gelling, G. E. Grigore, L. Sedek, A. E. Bras, G. Gaipa, E. Sobral da Costa, M. Novakova, E. Sonneveld, C. Buracchi, T. de Sá Bacelar, J. G. Te Marvelde, A. Trinquand, V. Asnafi, T. Szczepanski, S. Matarraz, A. Lopez, B. Vidriales, J. Bulsa, O. Hrusak, T. Kalina, Q. Lecrevisse, M. Martin Ayuso, M. Brüggemann, J. Verde, P. Fernandez, L. Burgos, B. Paiva, C. E. Pedreira, J. J. M. van Dongen, A. Orfao, V. H. J. van der Velden, Automated database-guided expert-supervised orientation for immunophenotypic diagnosis and classification of acute leukemia, *Leukemia* **32**, 874–881 (2018).

Supplemental Table 1. Distribution of different cell populations in normal/reactive PB samples according to age.

Cell population	Age Groups			All ages n=201
	0-5y n=88	6-15y n=33	>16y n=80	
**Lymphocytes**	29%-78%	24%-64%	187%-48%	21%-713%
B cells	2.5%-16%	2.6%-9.0%	0.96%-5.3%	1.2%-15%
T cells	18%-56%	17%-45%	12%-39%	15%-52%
NK cells	2.0%-11%	2.0%-16%	1.9%-9.7%	2.0%-11%
**Eosinophils**	0.53%-5.6%	0.29%-6.2%	0.44%-3.5%	0.44%-5.5%
**Neutrophils**	11%-60%	21%-67%	40%-72%	15%-68%
**Monocytes**	4.3%-13%	2.6%-13%	4.4%-12%	4.2%-12%
**Unspecified nucleated cells**	0.19%-1.3%	0.27%-1.7%	0.30%-1.3%	0.24%-1.3%

Results expressed as range 5th-95th of the percentile rank. Data are derived from 201 PB of healthy donors previously evaluated using the EuroFlow PIDOTtube (van Dongen et al, Frontiers Immunology 2019).

Supplemental Table 2. Distribution of different cell populations in normal/reactive BM samples according to age.

Cell population			Age Groups			
			0-5y n=13	6-15y n=10	>16y n=21	All ages n=44
**Lymphocytes**			13%-43%	6.1%-28%	9.8%-25%	10%-40%
	B cells		4.6%-37%	1.9%-16%	1.4%-8.6%	1.6%-37%
		CD34+ B cells	0.76%-5.1%	0.05%-1.9%	0.11%-1.0%	0.11%-4.7%
		CD34- B cells	3.9%-33%	1.8%-14%	1.2%-7.6%	1.5%-32%
	T cells		1.3%-16%	3.3%-11%	5.7%-21%	2.9%-18%
	NK cells		0.34%-3.2%	0.33%-1.2%	0.42%-5.3%	0.36%-3.7%
**CD34+ myeloid precursors**			0.86%-3.4%	0.25%-2.2%	0.27%-1.6%	0.30%-2.4%
**Eosinophils**			0.52%-5.2%	0.58%-2.8%	0.08%-3.8%	0.54%-3.8%
**Neutrophils**			36%-73%	48%-83%	56%-76%	40%-79%
**Monocytes**			3.3%-7.4%	1.4%-6.3%	1.9%-8.0%	2.0%-7.1%
**Nucleated red cells**			0.79%-20%	4.1%-19%	4.2%-20%	3.7%-20%
**Unspecified cells**			0.02%-2.11%	0.3%-0.83%	0.27%-1.29%	0.27%-1.57%

Results expressed as range 5th-95th of the percentile rank

Supplemental Table 3. Bone marrow (BM) and peripheral blood (PB) samples used for the validation of the AGI tool.

Reagents	Sample type	Number	Category	Gender (M/F)	Age
**EuroFlow**	PB	43	normal	18/25	37 (0-81)
**EuroFlow**	BM	31	normal	17/12[a]	12 (1-67)
**EuroFlow**	PB	13	AML	8/5	56 (2-75)
		11	B-ALL	5/6	4 (1-67)
		10	T-ALL	4/6	11 (1-62)
**EuroFlow**	BM	19	AML	14/5	59 (4-92)
		27	B-ALL	13/14	8 (2-79)
		10	T-ALL	9/1	14 (3-51)
		5	MPAL	3/1[b]	15 (5-17)
**OneFlow**	PB	15	10 normal, 2 B-ALL, 3 T-ALL	7/8	26 (1-53)
	BM	16	6 normal, 2 AML, 6 B-ALL, 2 T-ALL	6/10	10 (3-67)
**Cytognos**	PB	19	11 normal, 2 AML, 3 B-ALL, 3 T-ALL	8/11	34 (1-63)
	BM	40	8 normal, 9 AML, 20 B-ALL, 3 T-ALL	20/20	10 (1-92)

M=male, F=Female. Age shown as median with range between brackets

[a] Gender unknown for 2 cases;[b] unknown for one case

Infiltration by blast cells (median [min-max]) was 72%[0,3-98] in peripheral blood and 86%[2-100]) in bone marrow.

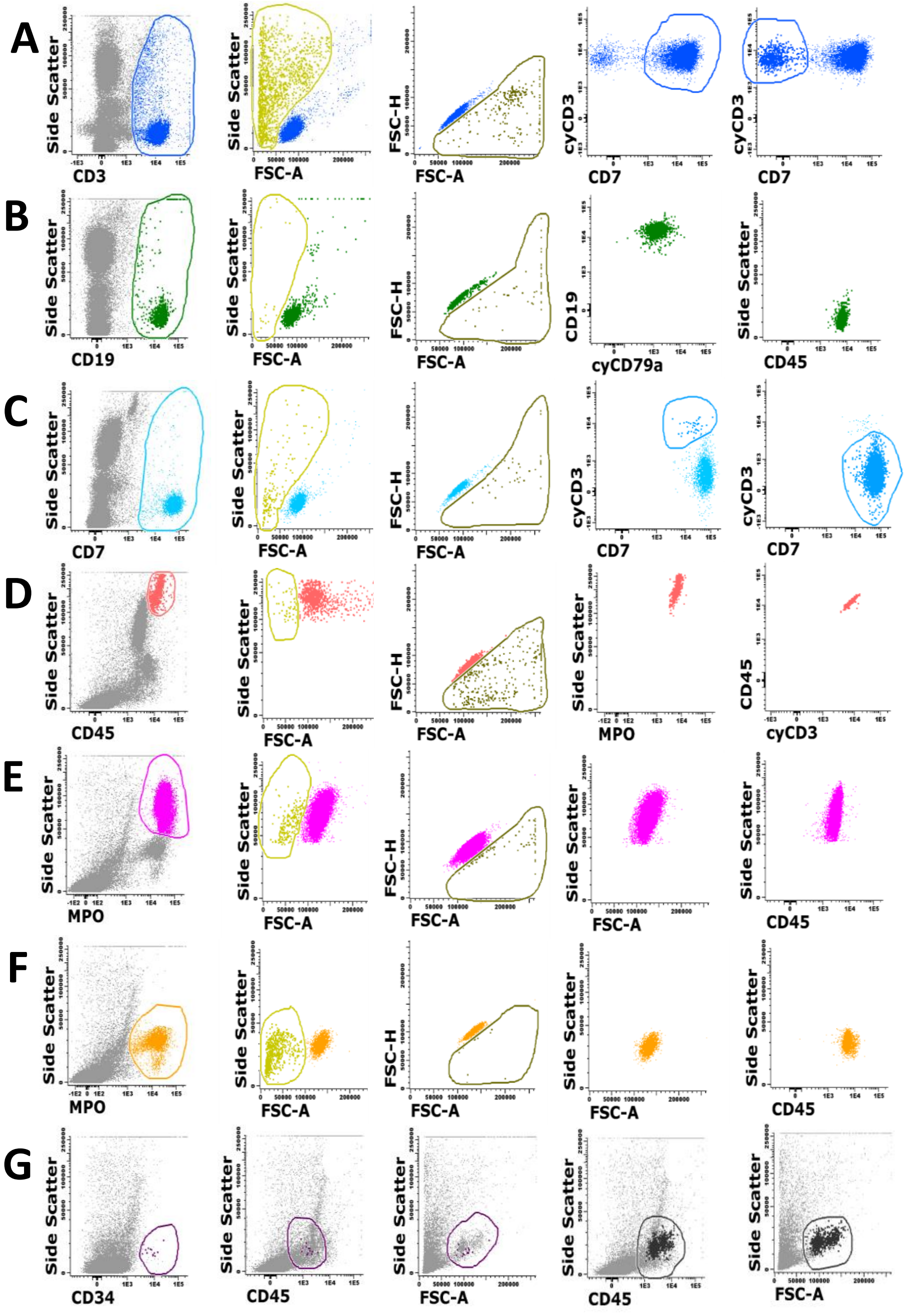

Supplemental Figure 1. Sequential gating strategy (A to G) for ALOT PB database cases.
Identification using the main lineage marker, debris and doublets exclusion (except panel G) and further subdivision using specific markers (if available)
A) T cells, B) B cells, C) NK cells, D) Eosinophils, E) Neutrophils, F) Monocytes, G) CD34 positive precursors and unspecified nucleated cells. The order for each analysis is left to right and A to G (first general gate on the major lineage marker, followed by debris and doublets exclusion and then subdivision within the cellular compartment).

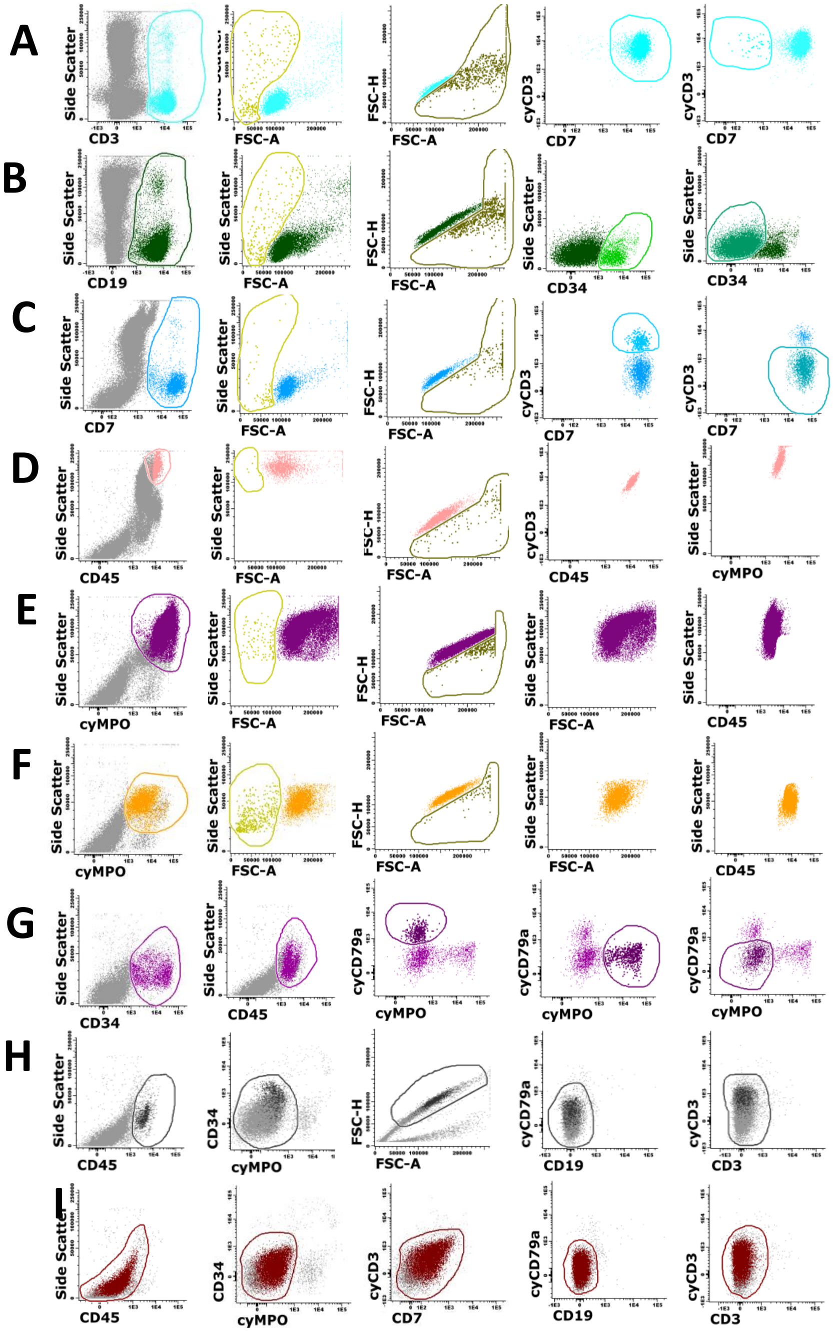

Supplemental Figure 2. Sequential gating strategy (A to I) for ALOT BM database cases.

Identification using the main lineage marker, debris and doublets exclusion (except panel G) and further subdivision using specific markers (if available)

A) T cells, B) B cells, C) NK cells, D) Eosinophils, E) Neutrophils, F) Monocytes, G) CD34 positive precursors, H) unspecified nucleated cells and I) Nucleated red cells. The order for each analysis is left to right and A to G (first general gate on the major lineage marker, follwed by debris and doublets exclusion and then subdivision within the cellular compartment).

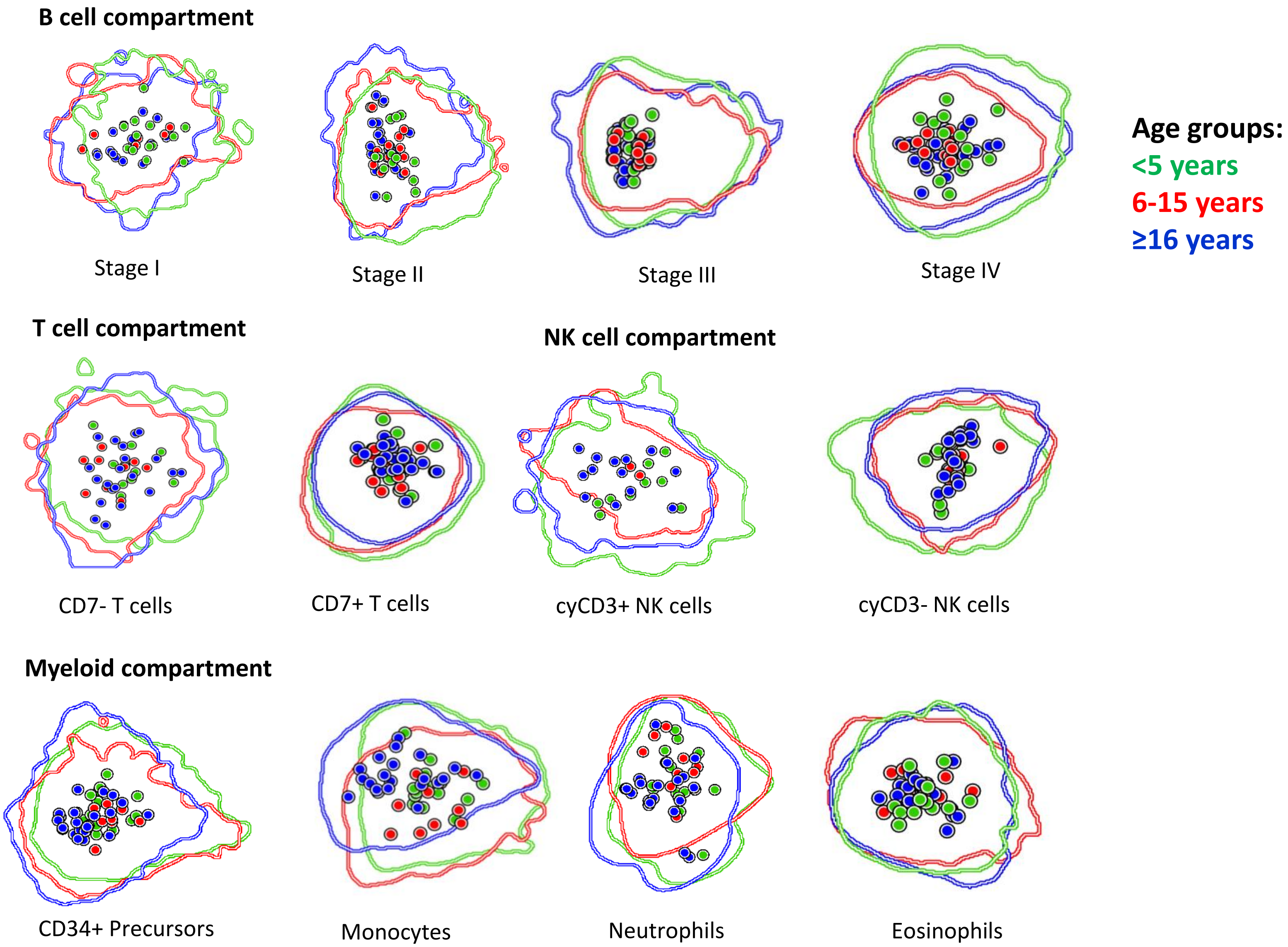

Supplemental Figure 3. Multidimensional (2SD PC based) views showing similar phenotypes between the age groups

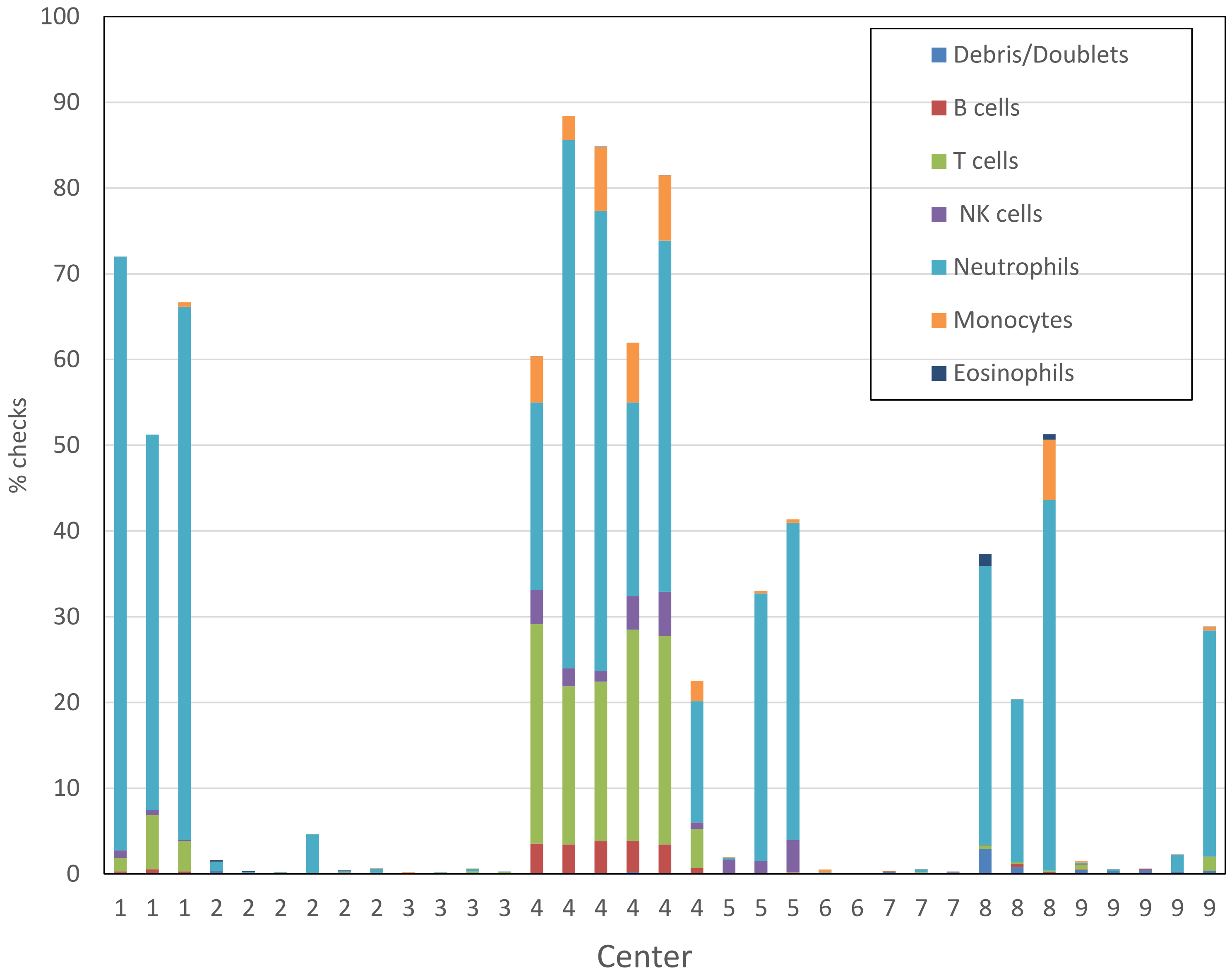

Supplemental Figure 4. Percentage of checks in normal peripheral blood samples analyzed by the ALOT AGI tool. Percentage of checks differed per center, mainly due to variations in the percentage of neutrophils that had to be checked.

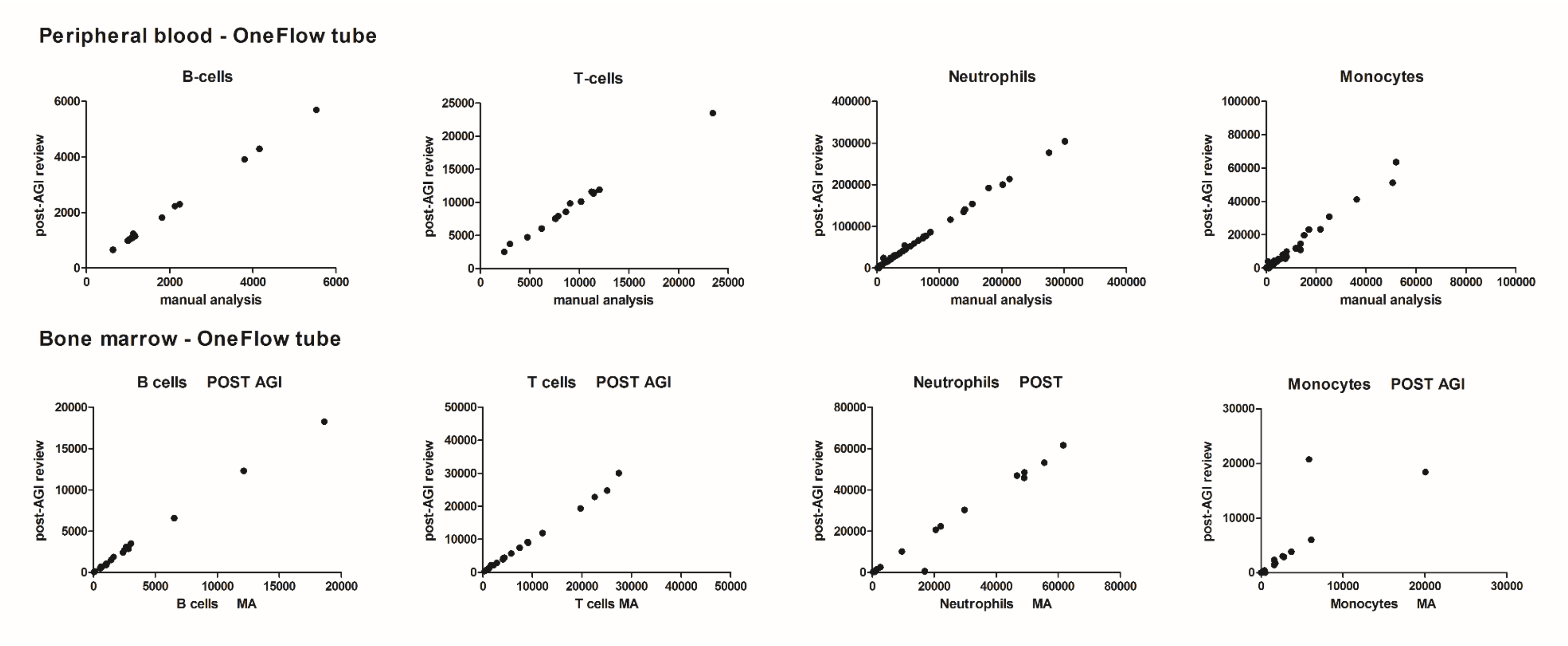

Supplemental Figure 5. Correlations between number of events for different leukocyte subsets present in peripheral blood (upper row) and bone marrow (lower row) as analyzed by manual analysis or AGI tool using the OneFlow tube.

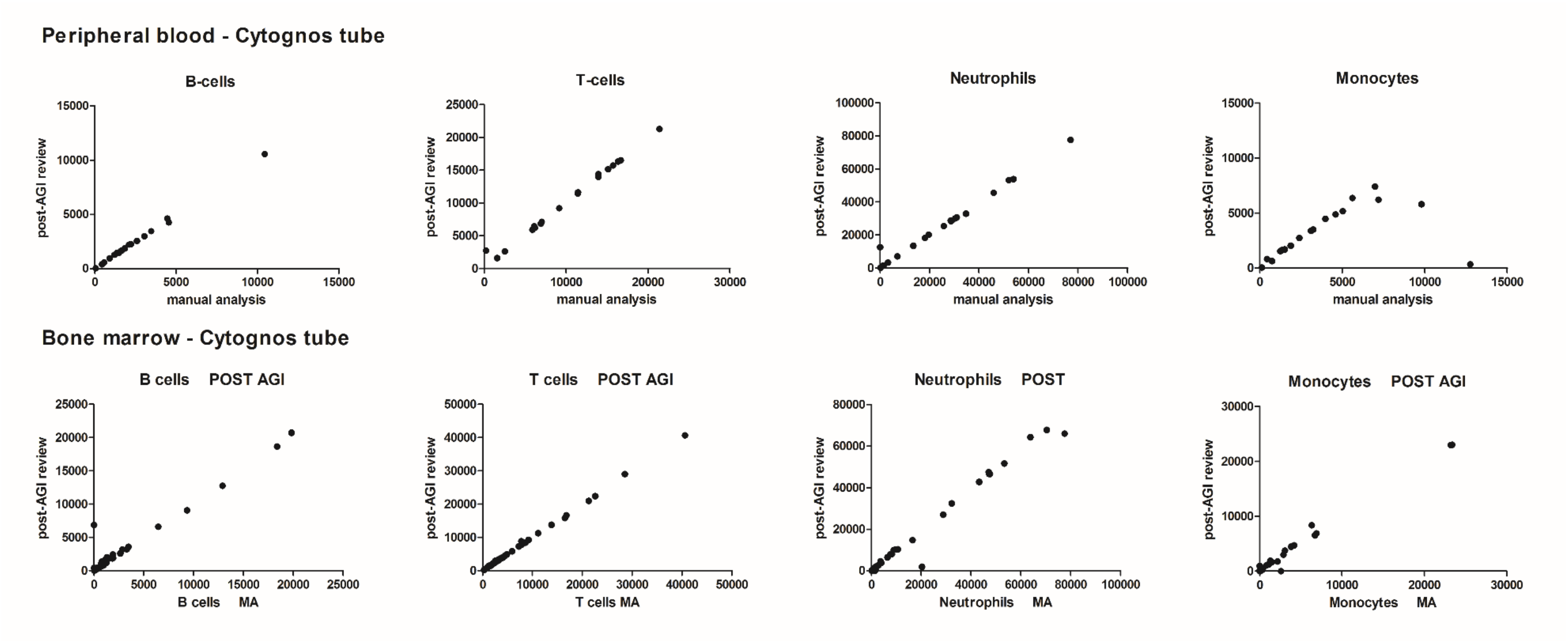

Supplemental Figure 6. Correlations between number of events for different leukocyte subsets present in peripheral blood (upper row) and bone marrow (lower row) as analyzed by manual analysis or AGI tool using the Cytognos mix.

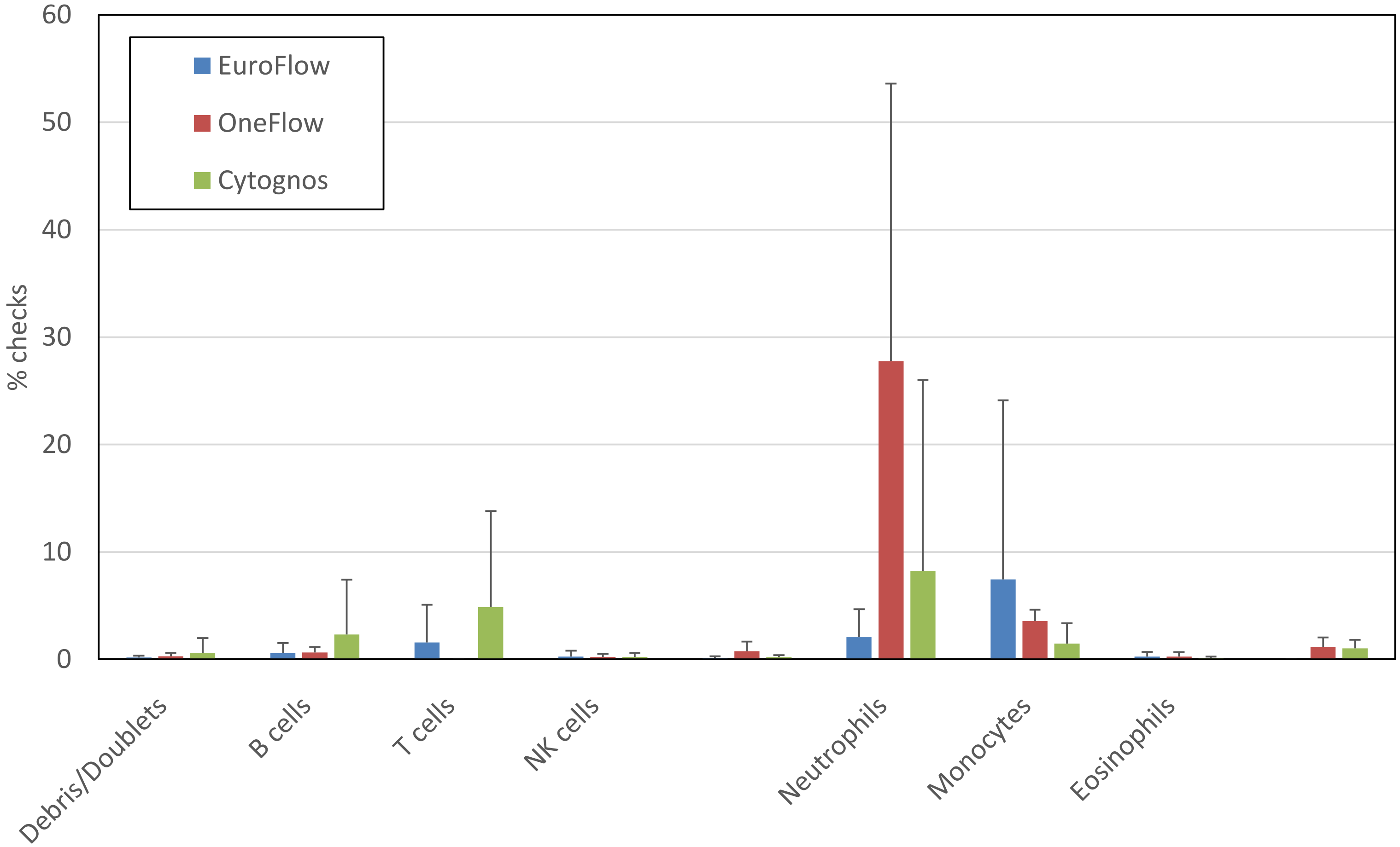

Supplemental Figure 7. Percentage of checks in six paired normal bone marrow samples either analyzed using the EuroFlow reference reagents (EuroFlow), using the OneFlow tube (OneFlow), or using the Cytognos reagents.

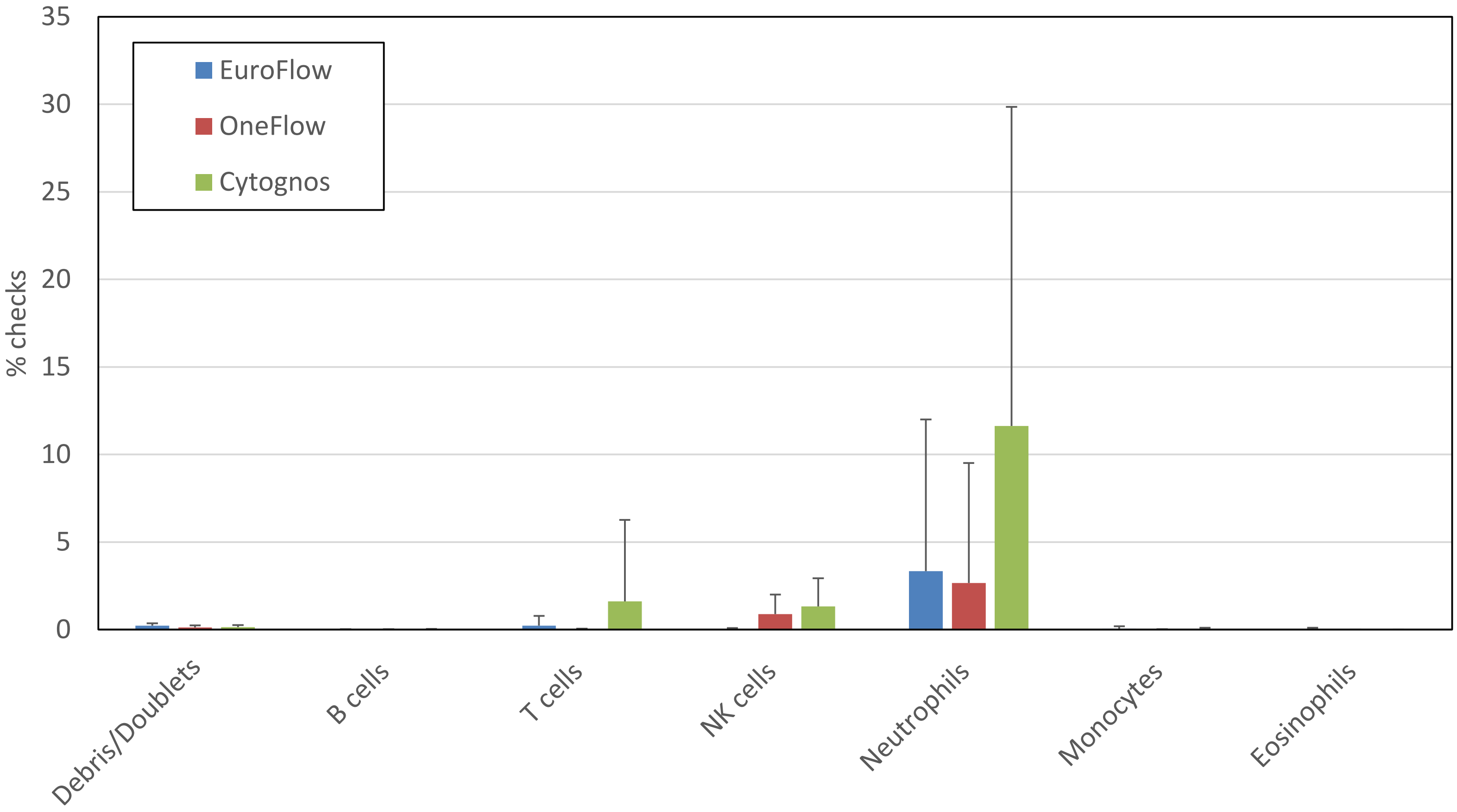

Supplemental Figure 8. Percentage of checks in nine paired normal peripheral blood samples either analyzed using the EuroFlow reference reagents (EuroFlow), using the OneFlow tube (OneFlow), or using the Cytognos reagents.

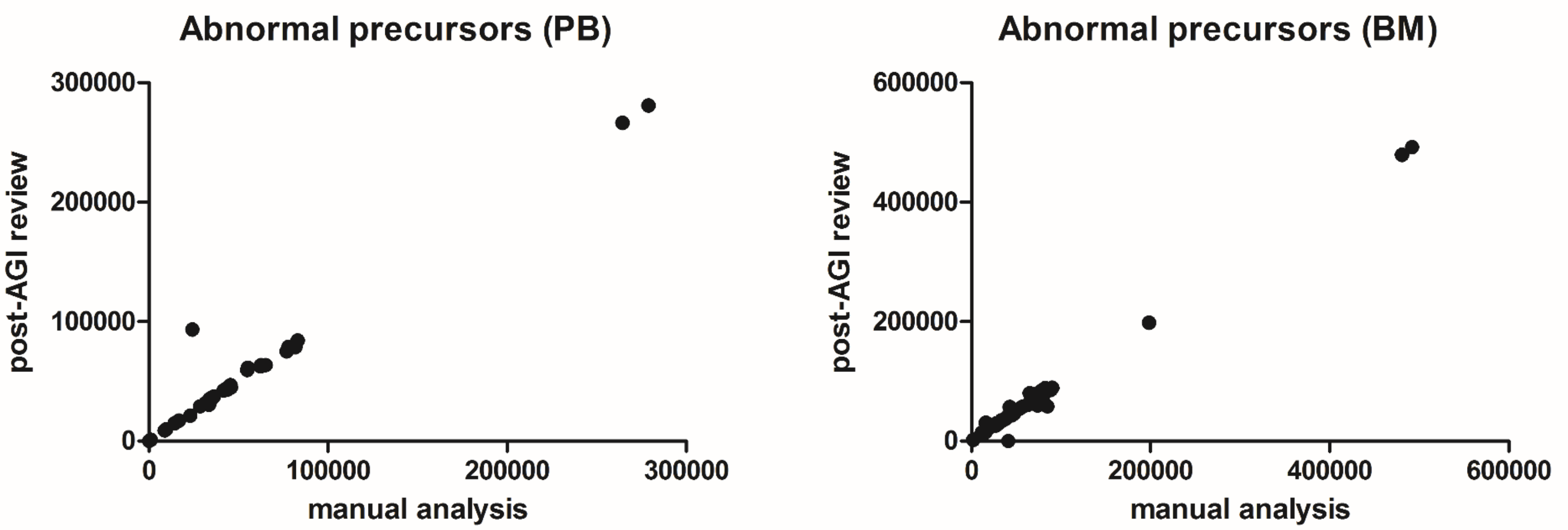

Supplemental Figure 9: correlation of leukemic cells as identified by manual analysis and the AGI tool

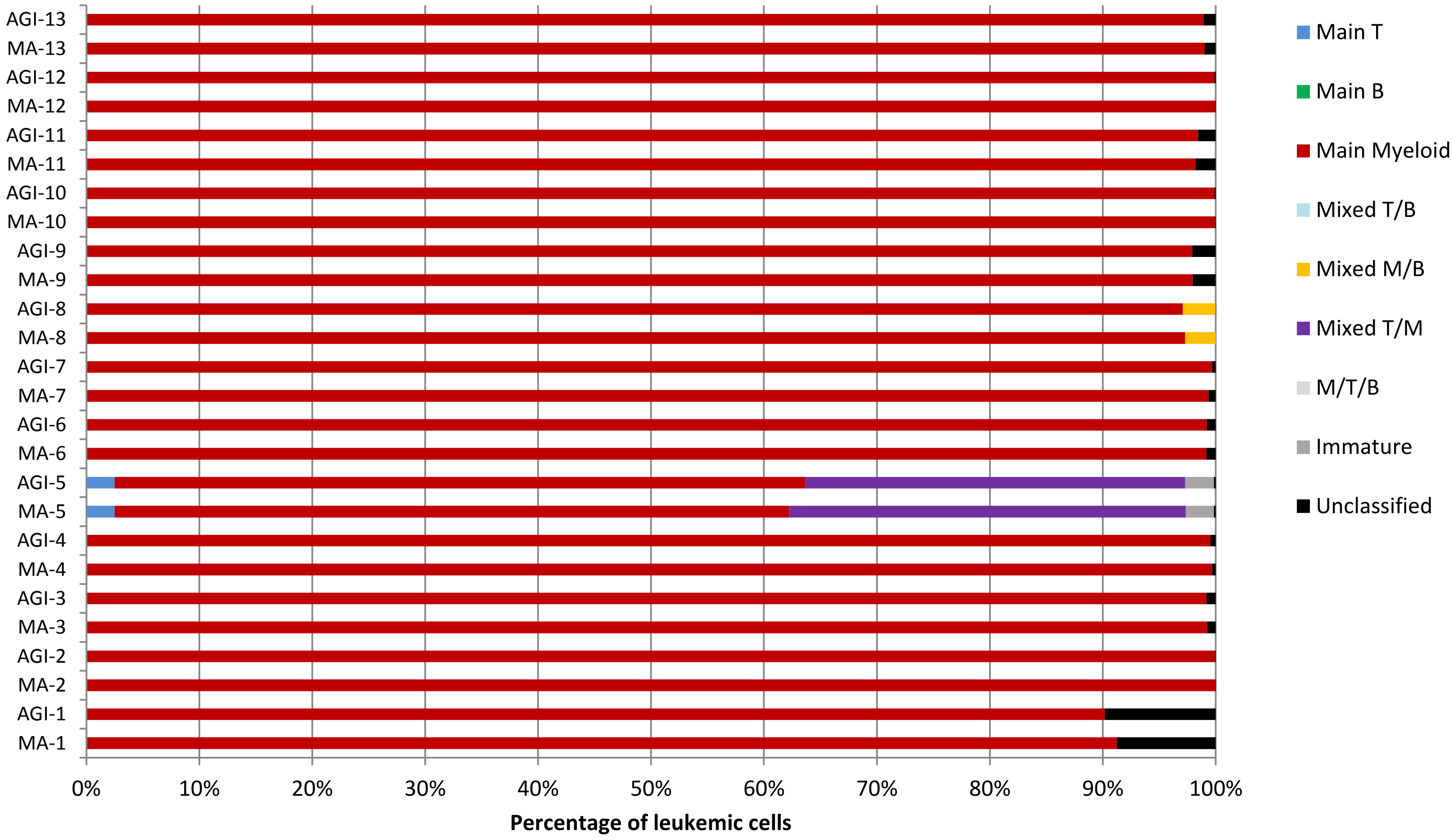

Supplemental Figure 10A: Compass results after manual analysis or AGI tool – PB AML

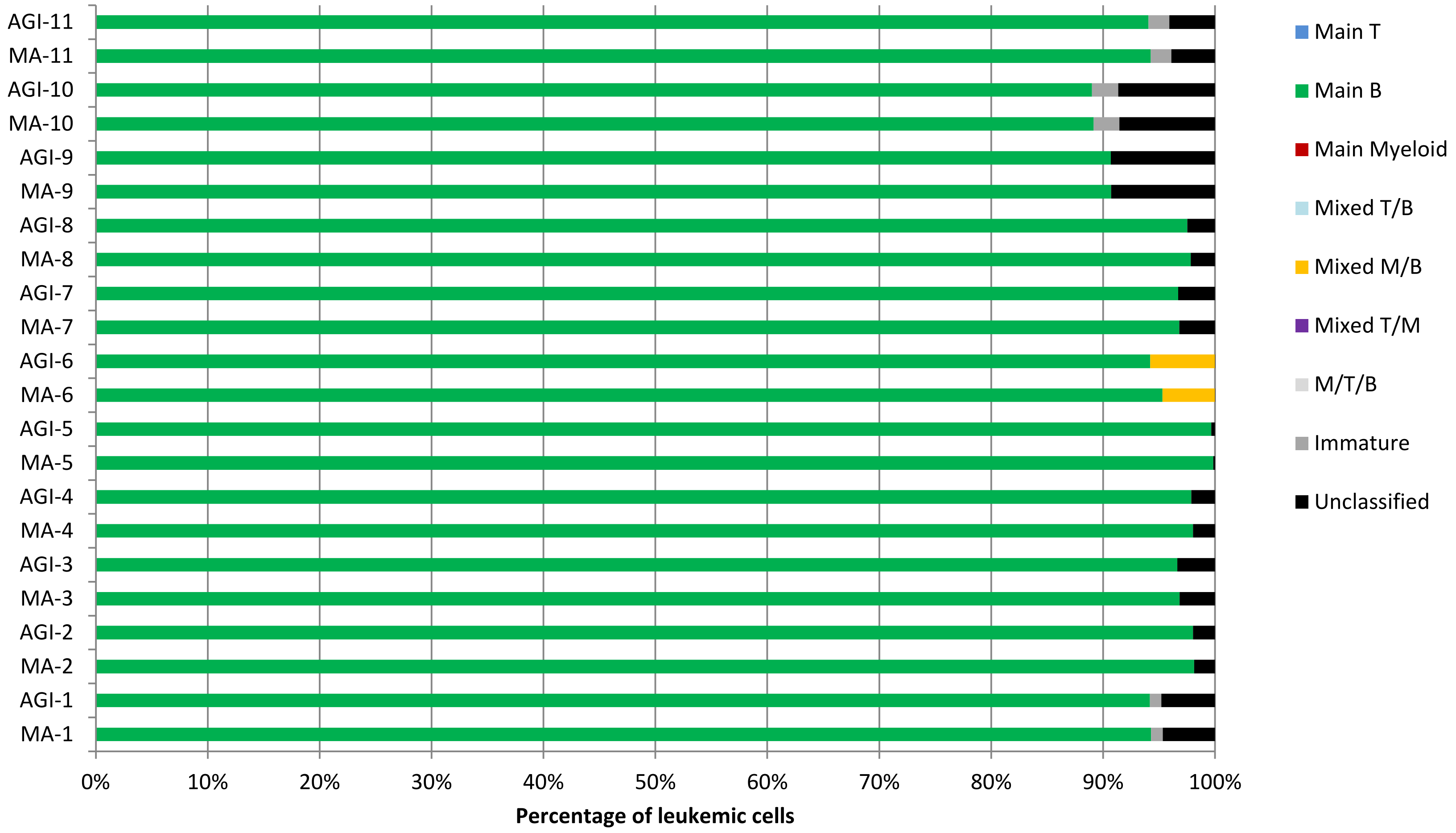

Supplemental Figure 10B: Compass results after manual analysis or AGI tool – PB B-ALL

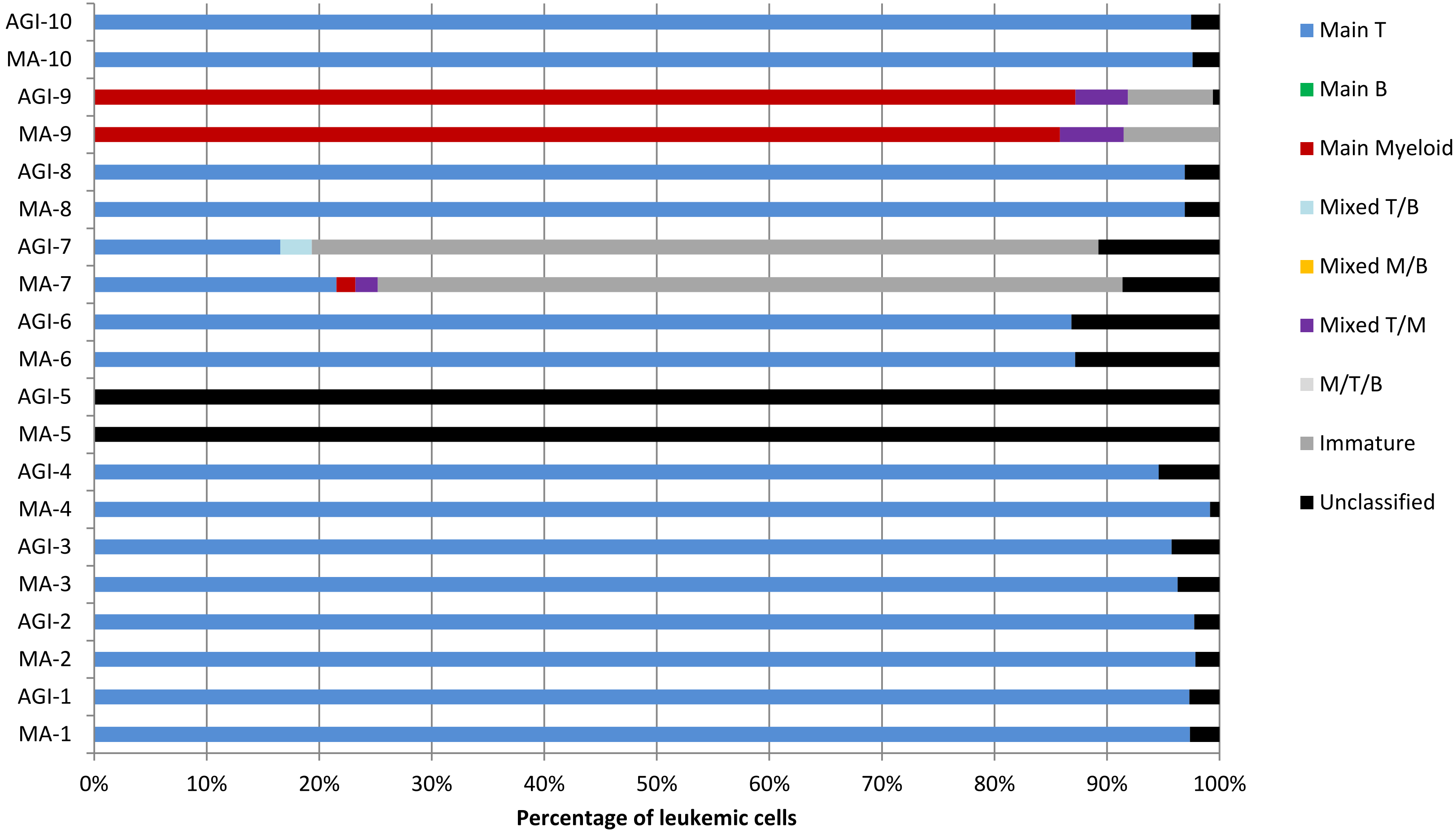

Supplemental Figure 10C: Compass results after manual analysis or AGI tool – PB T-ALL. Note: T-ALL9, which was CD7- and partically CyCD3-, was diagnosed as an Early T-cell precursor ALL. T-ALL5, positive for CyCD3, CD7 and MPO, was diagnosed as a relapsed T-ALL. Manual and AGI analysis yielded highly comparable results in all cases, including these two special cases.

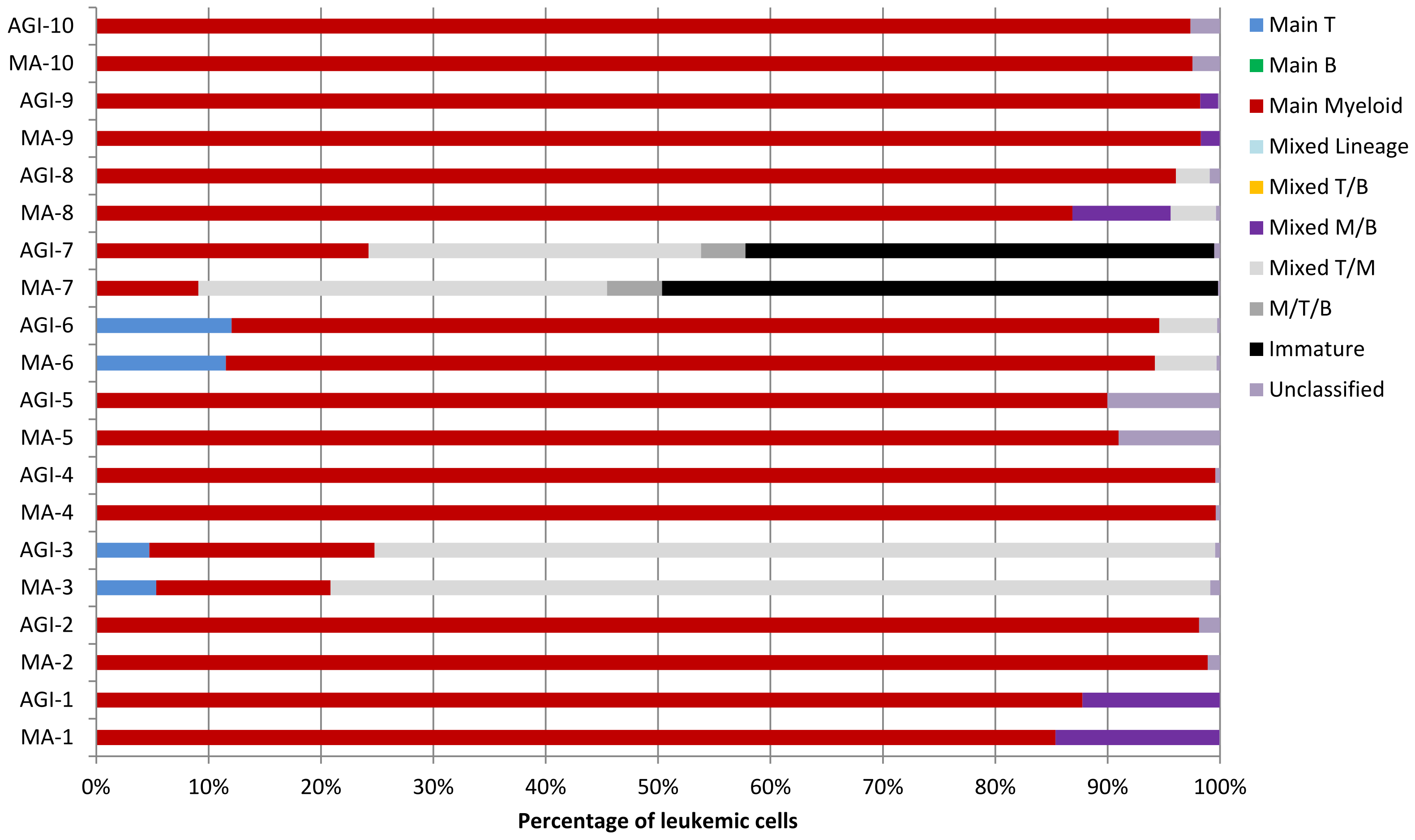

Supplemental Figure 11A: Compass results after manual analysis or AGI tool – BM AML

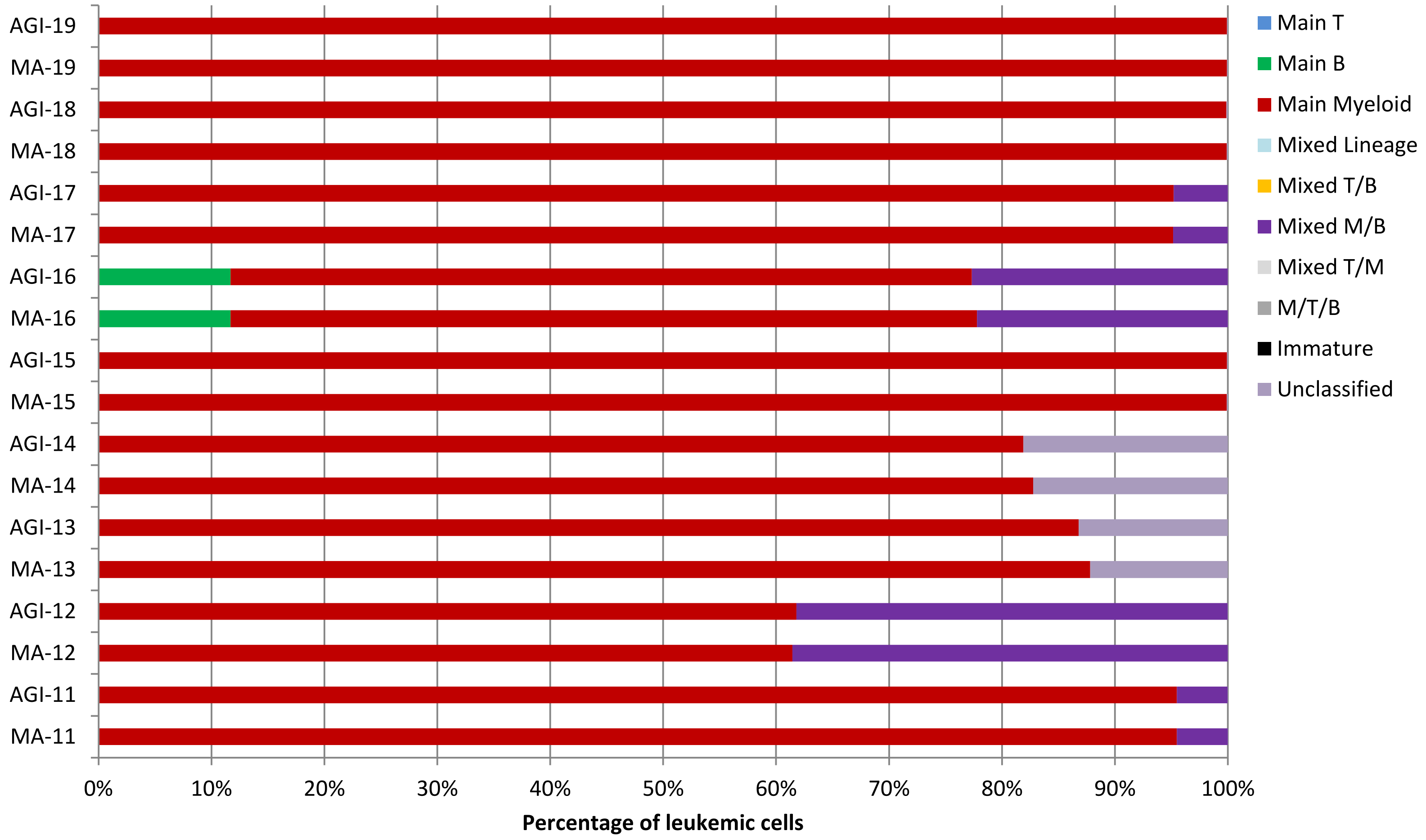

Supplemental Figure 11A (continued): Compass results after manual analysis or AGI tool – BM AML

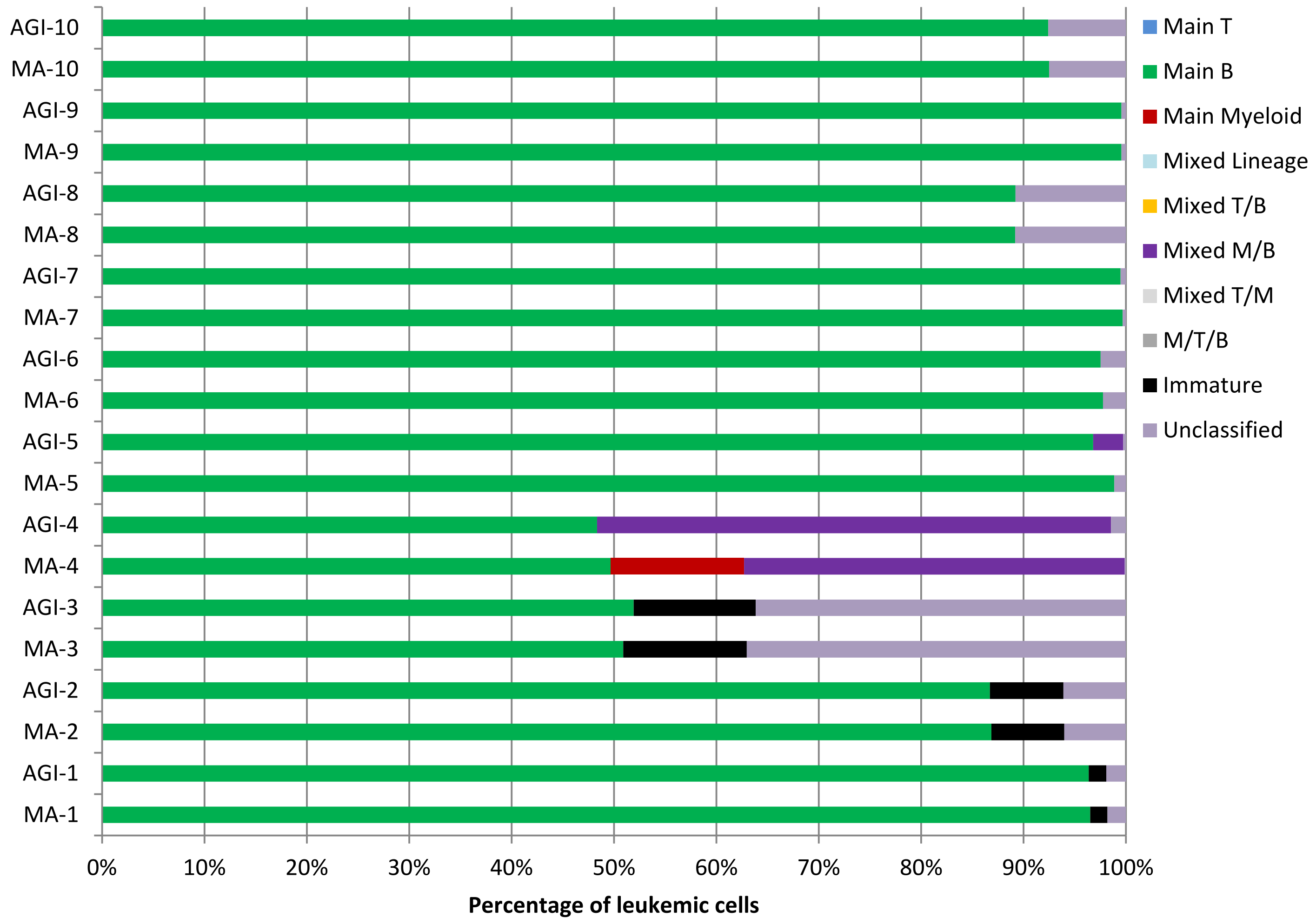

Supplemental Figure 11B: Compass results after manual analysis or AGI tool – BM BCP-ALL. Manual and AGI analysis yielded highly comparable results in all cases but one (BCP-ALL4), which was more variable but gave "non-typical" as result in both analyses.

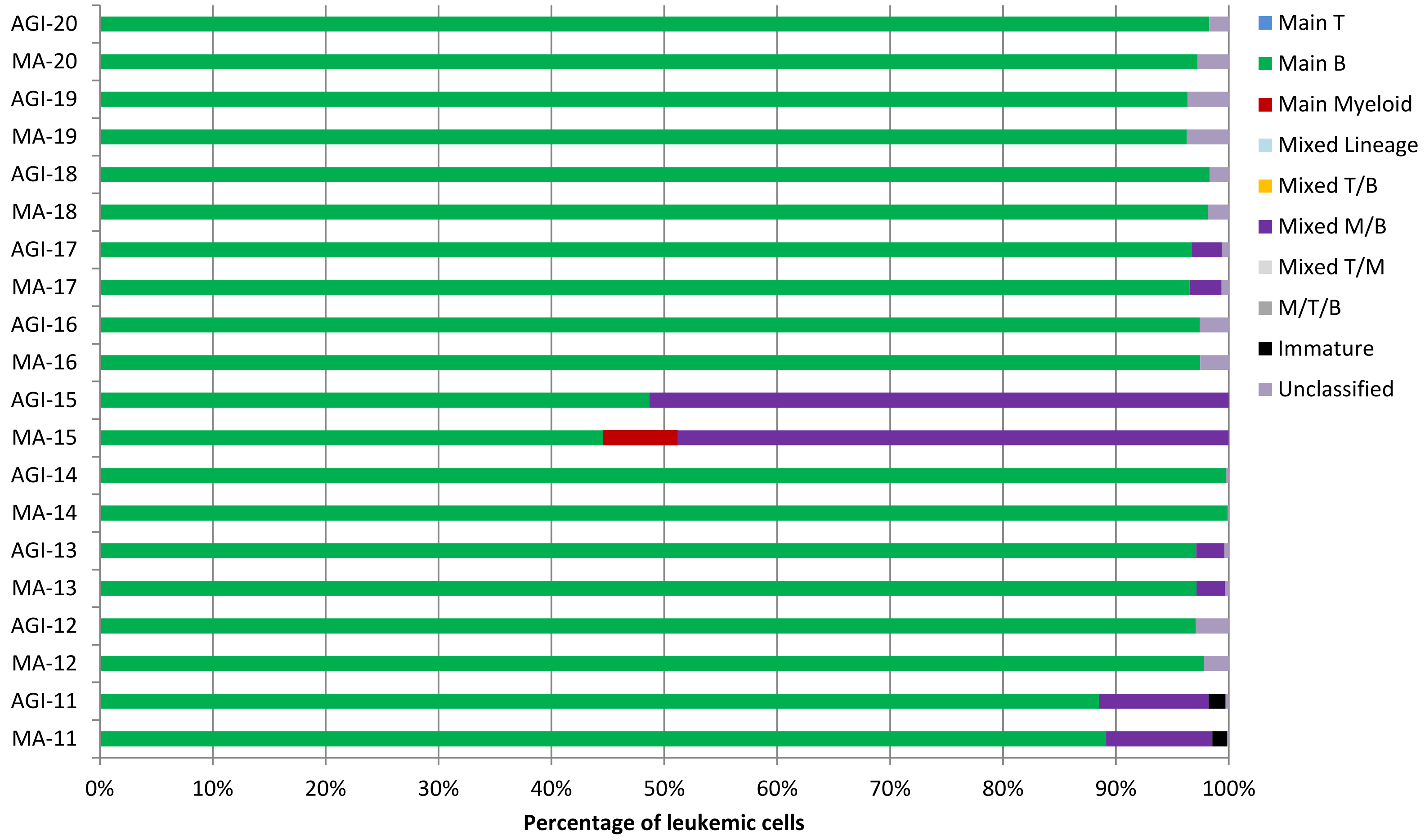

Supplemental Figure 11B (continued): Compass results after manual analysis or AGI tool – BM AML

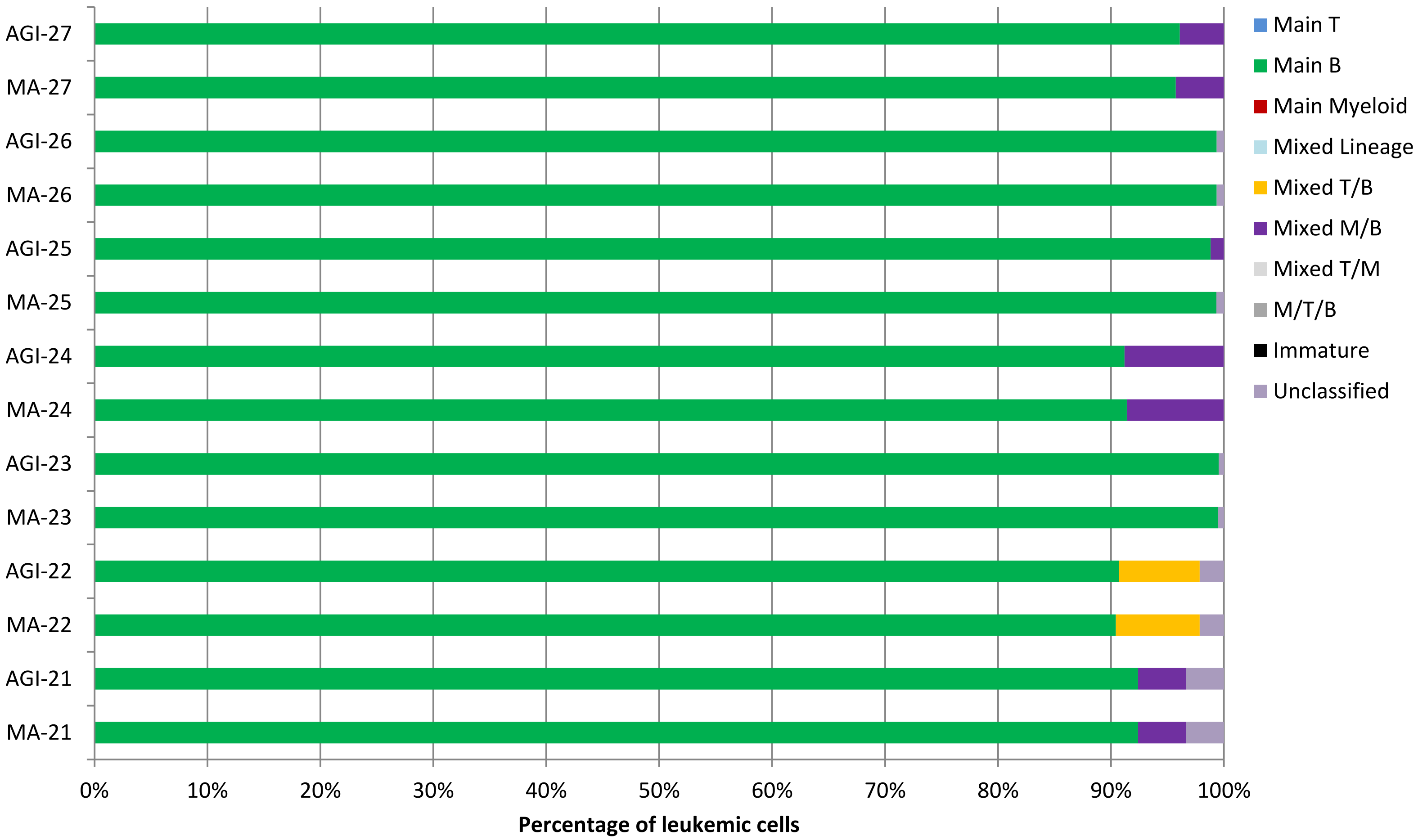

Supplemental Figure 11B (continued): Compass results after manual analysis or AGI tool – BM AML

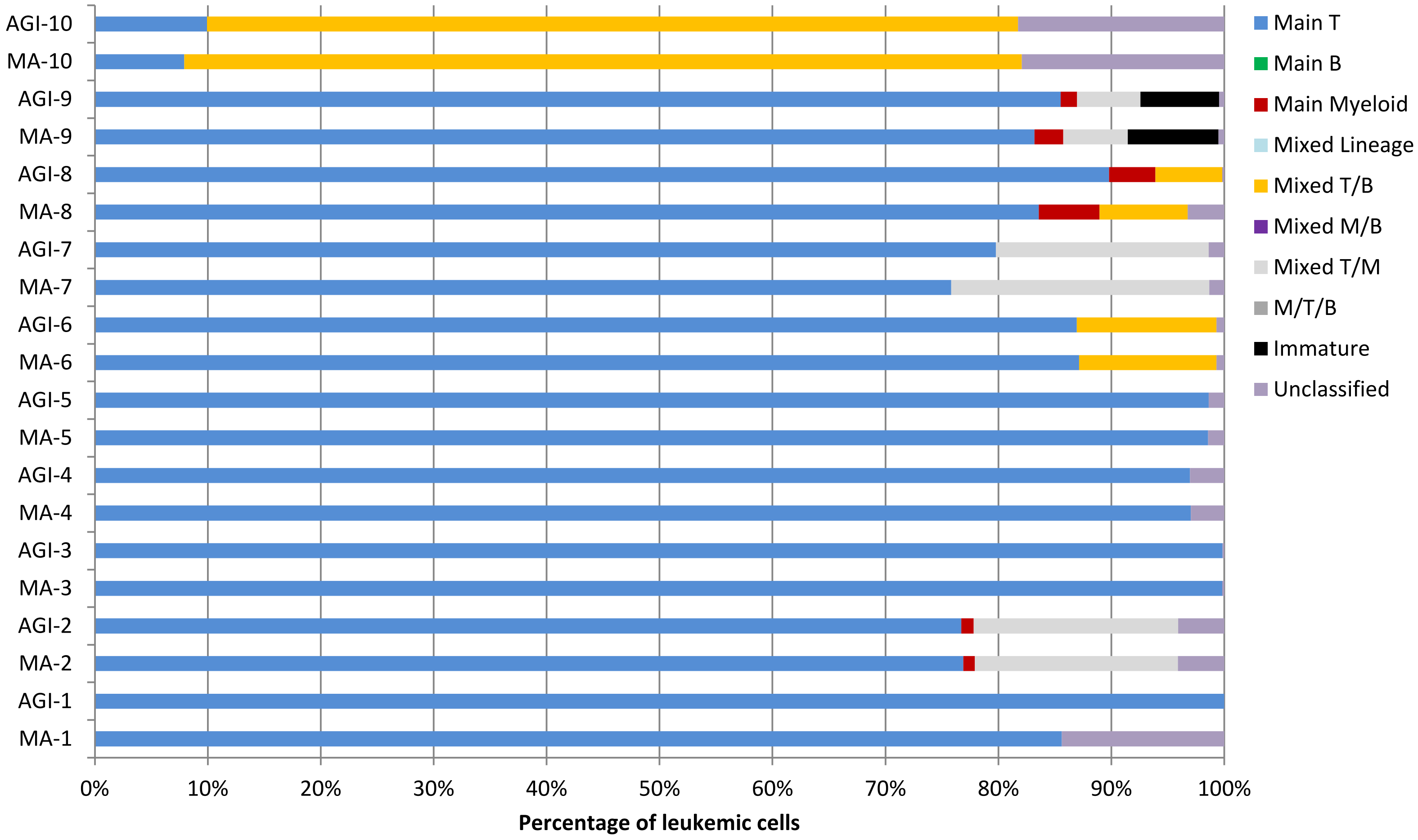

Supplemental Figure 11C: Compass results after manual analysis or AGI tool – BM T-ALL.

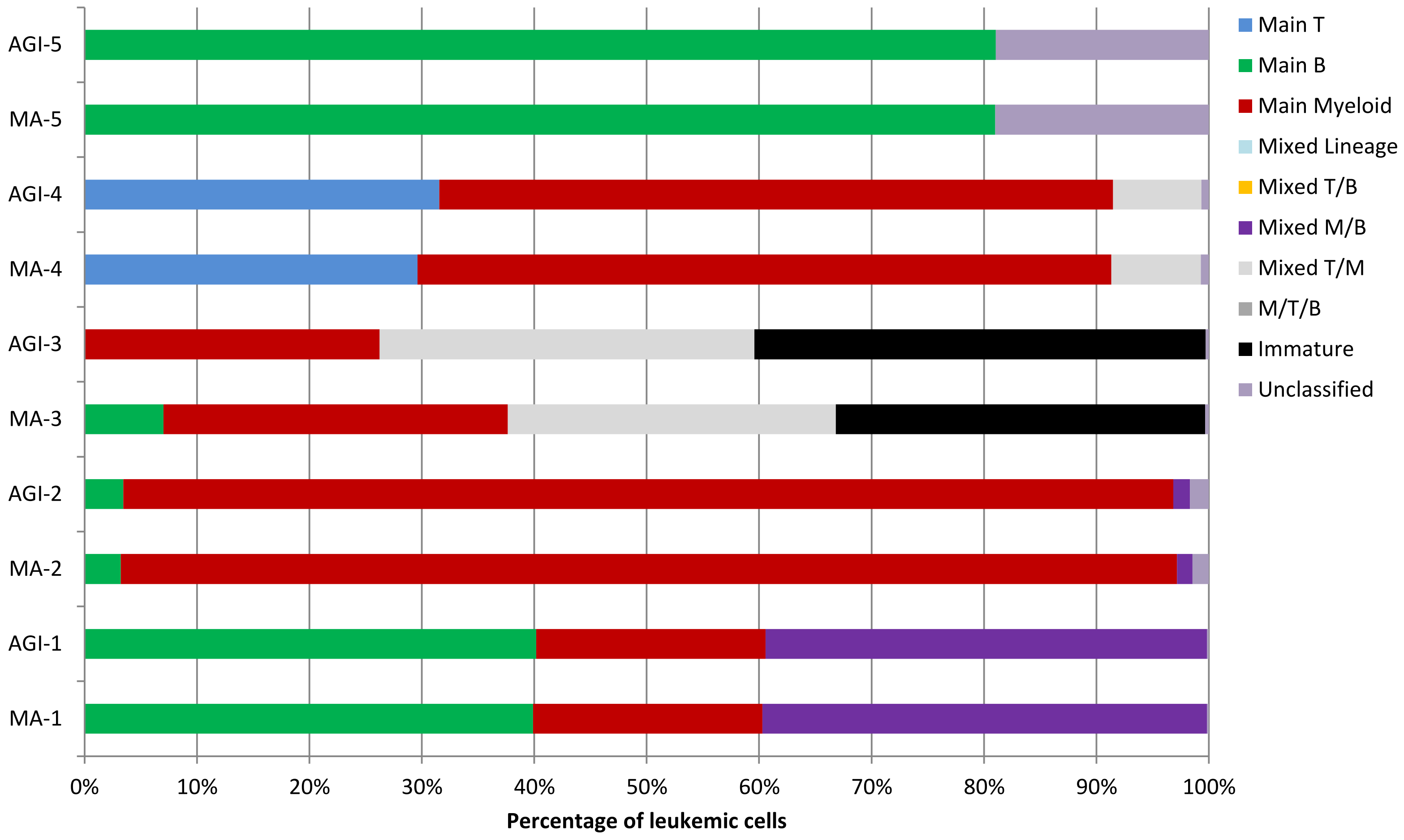

Supplemental Figure 11D: Compass results after manual analysis or AGI tool – BM MPAL

CELLULARITY (estimated based on total nucleated cells analyzed)

Reference range: < 6 years

Population	Frequency (%)	Reference (%)
**Lymphocytes**	**5.8**	(15.1 - 41.9)
**B cells**	**3**	(6.3 - 36.9)
**CD34- B cells**	**3**	(5.2 - 32.7)
T cells	2.4	(1.7 - 14.4)
NK cells	0.4	(0.36 - 3.1)
**Myeloid cells**	**0.78**	(43.6 - 79)
**CD34+ Myeloid precursors**	**0.1**	(0.88 - 3.1)
**Monocytes**	**0.047**	(3.3 - 7)
**Neutrophils**	**0.57**	(27.3 - 71.2)
**Eosinophils**	**0.052**	(0.57 - 4.5)
**Nucleated red cells**	**0.15**	(1.6 - 19.4)
**Abnormal precursors**	**93.2**	-
**B Lineage**	**91.4**	-

Absent populations: CD34+ B cells

IMMUNOPHENOTYPE DESCRIPTION OF ABNORMAL PRECURSORS

IMMUNOPHENOTYPE DESCRIPTION OF B LINEAGE BLASTS

$cyCD3^{-}$ $CD45^{lo/+}$(99.4%) $cyMPO^{-}$ $cyCD79a^{+/++}$(99.2%) $CD34^{+/++}$(93.2%) $CD19^{+/++}$(100%) $CD7^{-}$ $CD3^{-}$

*Reference population: normal B cells.

lo: low; hi: high.

Database normal cells have been used for the automated immunophenotypic description of the abnormal cells.

Supplemental Figure 12. A: Example of an automated report generated after the AGI tool and, if needed, the Compass tool is completed, first page

COMMENT

Infiltration by 91.4% of B lineage blasts (CD19$^{+/++}$(100%) cyCD79a$^{+/++}$(99.2%) CD34$^{+/++}$(93.2%)) detected.

Add additional comment:

CONCLUSION

Phenotypic findings compatible with infiltration by B acute lymphoblastic leukemia (B-ALL). It is recommended to perform further studies using the B-ALL panel.

Add additional conclusion:

Supplemental Figure 12. B.: Example of an automated report generated after the AGI tool and, if needed, the Compass tool is completed, second page
